# Impact of a Patient-Facing Enhanced Genomic Results Report to Improve Understanding, Engagement, and Communication

**DOI:** 10.1007/s10897-017-0176-6

**Published:** 2017-12-04

**Authors:** Janet L. Williams, Alanna Kulchak Rahm, Doris T. Zallen, Heather Stuckey, Kara Fultz, Audrey L. Fan, Michele Bonhag, Lynn Feldman, Michael M. Segal, Marc S. Williams

**Affiliations:** 1 Geisinger Genomic Medicine Institute, 26-20, 100 N. Academy Ave., Danville, PA 17822 USA; 2 Virginia Tech, Blacksburg, VA USA; 3 Penn State Hershey College of Medicine, Hershey, PA USA; 4 Department of Health, Philadelphia, PA USA; 5 Genome Medical, Inc., San Francisco, CA USA; 6 Chase Group, Ltd., Traverse, MI USA; 7 SimulConsult, Inc., Chestnut Hill City, MA USA

**Keywords:** Genetic testing reports, Rare disease, Clinical decision support- genomics, Mixed-methods, Patient-centered outcomes

## Abstract

“The objective of this study was to” test the effectiveness of an enhanced genomic report on patient-centered outcome domains including communication, engagement and satisfaction. “Study design utilized” a prospective, randomized, mixed-methods desctiptive study of a whole genome sequencing results report, GenomeCOMPASS™, that was accessed by providers through the electronic health record and by patients through the associated patient portal. “The study was set in” an integrated healthcare delivery system in central Pennsylvania. “Eighty-four” parents of 46 children with undiagnosed Intellectual Disability, Autism Spectrum Disorder and/or multiple congenital anomalies who had participated in a previous study offering whole genome sequencing for their affected child were invited to enroll. Fifty-two parents enrolled. Following a traditional genetics results informing visit, the study coordinator stratified families by diagnostic result and uninformative result and then randomized families within each group to an intervention arm to receive the GenomeCOMPASS™ report or to the usual care arm to receive a summary letter from the medical geneticist. A letter inviting enrollment included a baseline survey, which once returned, constituted enrollment. Surveys were administered at 3 months post-genetics visit. At 6 months, the usual care arm crossed over to receive the intervention and were administered an additional survey at 3 months. Qualitative interviews were conducted following survey completion to augment the survey data regarding the patient centered outcomes of interest. Patient reported outcomes including communication, engagement, empowerment and satisfaction. In the intervention arm, GenomeCOMPASS™ reports were released to 14 families (*N* = 28 parents) and of those 21 (75%) returned 3 month surveys. In the usual care arm, 12 families (*N* = 24 parents) received usual care summary letters and of those 20 (83%) returned 3 month surveys. At crossover, GenomeCOMPASS™ reports were released to 20 individuals and 15 (75%) returned 3 month surveys. Qualitative interviews were conducted with 5 individuals. Use of the GenomeCOMPASS™ report was reported by this small group of parents to improve communication with providers and non-health professionals such as educators and therapists and led to increased engagement and high satisfaction. Providers and others involved in the children’s care also endorsed the report’s effectiveness. Reports that addressed negative findings, i.e. uninformative results, were not found to be useful. Although the number of users was small, this study supports that customizable template reports may provide a useful and durable source of information that can support and enhance the information provided by genetics professionals in traditional face-to-face encounters. Trial registration: Clinicaltrials.gov (Record 2013–0594).

**Electronic supplementary material:**

The online version of this article (10.1007/s10897-017-0176-6) contains supplementary material, which is available to authorized users.

## Introduction

Genetic disorders, while individually rare are collectively common. Most are chronic and impact patients and their families for life. The challenge for patients and their providers is having ready access to the information that is necessary for appropriate management and coordination of care. With common disease, evidence-based professional practice guidelines allow providers access to best practices that can inform shared decision-making with their patients. In genetic disease, guidelines are available only for a handful of the more common rare diseases. (Levy et al. 2008) Fully functional electronic health records (EHR) with embedded point of care information resources and clinical decision support (CDS) have been shown to aid providers and (in the few situations studied) patients. (Lobach et al. 2012) This approach has been used to improve the use of genomic information in clinical care. (Welch and Kawamoto 2013) Most CDS implementations to date have involved pharmacogenomics which is more straightforward than rare genetic disease although CDS has also been applied to family history and genetically guided cancer care. As of 2013, nine randomized controlled trials utilizing CDS for genomic medicine have been conducted, of which seven showed positive results. (Welch and Kawamoto). All of the tested CDS interventions were directed for providers. To date, no genomic CDS has been directed at patients, or patients and providers through the EHR. Direct to consumer (DTC) genetic testing companies have extensive experience developing informational materials for the public, but these are stand-alone and do not interact with EHRs. In general, the DTC companies do not perform diagnostic testing for rare disease.

**Fig. 1:**
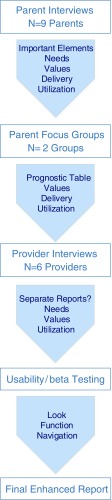
Flow diagram from development to deployment of the enhanced genomic report

To address this need, we envisioned a patient-facing genomic test report designed by patients for patients. (see Fig. 1) In previously published work, we described the development of an enhanced genomic test report informed by the perspectives of parents and providers through user-centered design principles. (Stuckey et al. 2015; Williams et al. 2016) It is important to note, that the enhanced genomic report is not meant to replace the laboratory results report, rather it presents the genomic result from the laboratory report in language accessible by patients, family members and non-genetics providers.

Application interfaces are increasingly used to lower barriers to implementation of CDS and genomic examples are emerging. (Swaminathan et al. 2016) In a manuscript that is submitted and in review, Williams described the technical development of GenomeCOMPASS™, a software application that enables the presentation of the enhanced genomic report through an interface accessible in the EHR. This allows the provider to view the GenomeCOMPASS™ report during a visit encounter while the patient in is the room and allows the patient to access the report anywhere via the patient portal associated with the EHR. We hypothesized that this new form of functional genetic test report, presented to providers in the EHR, and with a patient accessible view, would improve shared decision-making, enhance condition-specific management, and improve outcomes from both the patient/family and provider perspectives. To test this hypothesis, we performed a prospective randomized mixed-methods descriptive study of the GenomeCOMPASS™ report in a population of patients and families participating in a study that used whole genome sequencing (WGS) for children with undiagnosed intellectual disability. In this paper, we present the results of the GenomeCOMPASS™ report on patient-centered outcomes including communication, engagement, empowerment and satisfaction.

## Methods

**Fig. 2:**
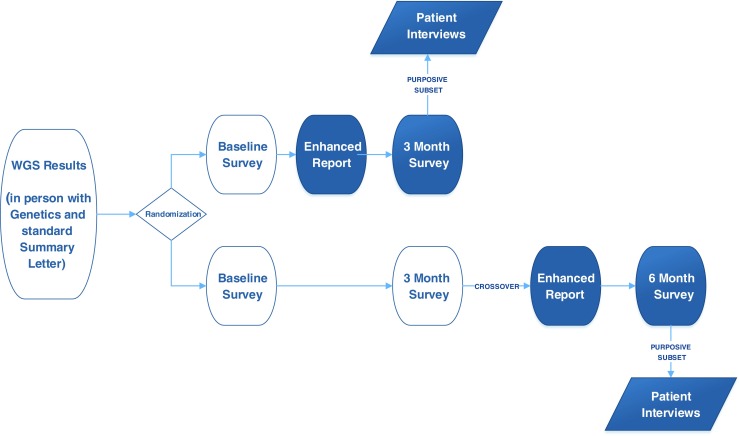
Effectiveness study design

Utilizing an Explanatory Sequential mixed-methods design (Creswell 2015), we conducted a prospective randomized pre-post asymmetric crossover study paired with post-intervention qualitative evaluation to study the effectiveness of the GenomeCOMPASS™ report on patient-centered outcomes focused on communication, engagement and satisfaction (see Fig. 2). The study was approved by the Geisinger Institutional Review Board and registered at clinicaltrials.gov (Record 2013–0594). For this study, the GenomeCOMPASS™ report is also referred to as the “enhanced genomic report”.

Subjects for this study included parents (*N* = 84) of children with rare, unexplained Intellectual Disability (ID), Autism Spectrum Disorder (ASD) and/or multiple congenital anomalies enrolled in a separate Whole Genome Sequencing (WGS) Research Study (utilizing a study within a study approach). Both parents were required to participate in the WGS study for purposes of genomic results interpretation, which offered the opportunity in the current study to capture potential differences in response to the enhanced genomic report between fathers and mothers. After the return of WGS results via a traditional in-person genetics visit, parents were stratified by genomic test result (diagnostic vs. uninformative) and then randomized (as couples within each group) to receive either the enhanced genomic report (intervention) or usual care. Randomization by couples was chosen recognizing that randomization at the individual level could have led to contamination and spillover if one member of the couple was in the usual care arm and the other was in the intervention arm. In order to test the impact of the enhanced genomic report regardless of test result, parent couples receiving a diagnostic result were evenly allocated to intervention or usual care arm, as were all parent couples who received an uninformative result on WGS. Parents and Investigators were blinded to the randomization.

The invitation to participate in this study was offered at the genetics visit and followed with a letter that provided a description of this study and a copy of a baseline survey. Parents were assured that participation was voluntary and would not impact the care of their child or their own healthcare. Parents who completed the baseline survey were then enrolled in the effectiveness study and provided access to the intervention (the enhanced genomic report) or usual care based on the assigned prior randomization (Fig. 2).

In this study, usual care consisted of a summary letter composed by the medical geneticist that was sent following a typical genomics results return session. All sessions were conducted by the same medical geneticist and one of two genetic counselors. The summary letter did not include a copy of the genome sequencing laboratory report. This usual care arm is representative of current genetic practice in which laboratory reports are generally returned only to the ordering provider and the results are discussed with the patient/family during a clinical visit.

The intervention arm consisted of usual care (with the summary letter sent by the medical geneticist) and followed with the release of the enhanced GenomeCOMPASS™ report to the child’s parents through the online patient portal.

**Fig. 3:**
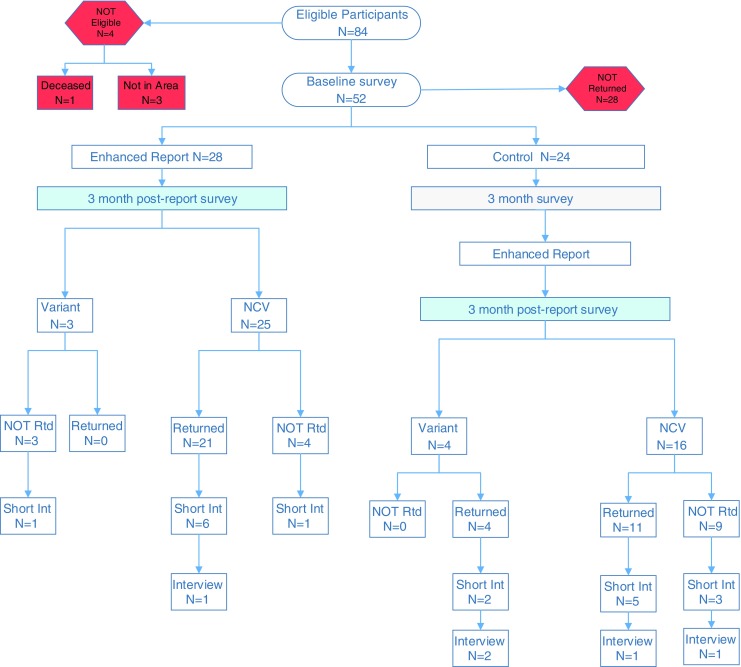
Participant flow diagram. CV = Causal Variant/Diagnostic: genetic cause to explain the child’s symptoms was found (diagnosis received). NCV – Non-Causal Variant/Uninformative: no genetic cause for the child’s symptoms was identified (no diagnosis received). Short Int = short interview: abbreviated interview to reduce missing data and enhance understanding of use/non-use of the enhanced report

As shown in Fig. 3, an asymmetric crossover arm presented the enhanced genomic report to all enrolled participants in the usual care arm.

Over the course of the study, individuals in the intervention arm completed two survey instruments (at baseline and at 3-months post-launch of the enhanced genomic report. Copies available in Online Resource 1,2). Individuals in the usual care/cross-over arm received three surveys (baseline; 3 months after summary letter was sent, and 3 months after release of the enhanced genomic report). Three-months post-launch of the enhanced genomic report (intervention or cross-over) parent participants were invited (via recruitment letter followed by phone call) to participate in an in-depth interview about using the enhanced report. Individuals with missing survey data or incomplete surveys were contacted by study staff for a short phone call interview to invite completion of the survey over the phone. The remaining participants in the study who did not complete surveys, still received the enhanced report at the conclusion of this study. All surveys were conducted via mail with the option to complete via telephone if desired; all telephone contacts were conducted by the study coordinator. Participants were provided a $25 gift card at the completion of each survey and interview.

Survey Measures are listed in Table 1 and included demographics, use of the EHR, use of resources, and a set of validated instruments including satisfaction, decision regret and other outcomes. Semi-structured interviews (Interview guide available in Online Resource 3.) were conducted by 1 genetic counselor with extensive qualitative experience (AKR) at 3 months post-intervention to further understand outcomes including utilization, impact, and unintended consequences of the enhanced genomic report. (see Table 2).

**Table 1:** Effectiveness study measures and timing with citations

Scale	Items	Measure	Baseline	3mos	6mos (crossover ONLY)
Adapted MICRA^a^	22	impact of genetic testing	x	x	x
Health literacy^b^	1	functional Health Literacy	x		
Numeracy^c^	4	numeracy	x		
Psychosocial Adjustment to Genetic Information (PAGIS)^d^ (Diagnostic Only)	10	certainty subscale – understanding of information	x	x	x
Health Information Orientation Scale (HIOS)^e^	8	health information preferences and engagement with sources	x		
General health^c^	6	general health and confidence caring for health/child’s health	x	x	x
Provider communication^c^	8	communication with provider and using internet w/ provider	x	x	x
Internet use and info seeking^c^	7	internet use, confidence w/ resources, use of other resources	x	x	x
Demographics	10	income, age, gender, education attainment	x		
Decision Regret^f^	6	decision regret for genetic testing child	x	x	x
Total questions	82			67	67

^a^Cella et al. 2002

^b^Chew et al. 2008

^c^Institute NC. Health Information National Trends Survey n.d.

^d^Read et al. 2005

^e^DuBenske et al. 2009

^f^Brehaut et al. 2003

**Table 2:** Effectiveness study outcomes with collection methods, sources, and measures

Outcome	Collection method	Measure	Timing of collection
Primary outcomes			
Utilization	COMPASS	Whether report was accessed and by whom	By 3 month survey
Satisfaction	Parent survey	3 survey questions	3 month post-intervention
Impact	Parent interview	Structured interview	3 months post-intervention
Demographics			
Literacy	Parent survey	Scale - HINTS	Baseline
Numeracy	Parent survey	Scale - HINTS	Baseline
Race ethnicity	Parent survey	Scale	Baseline
Secondary outcomes			
Decision regret	Parent survey	Scale – decision regret	Baseline, 3 months post- intervention
Report impact	Parent interview	Structured interview	3 months post-intervention
Communication	Survey and interviews	Scale – HINTS Structured interview	Baseline, 3 months post-intervention
Unintended consequences	Parent interview	Structured interview	3 months post-intervention

Processes to maximize completion of the baseline survey for enrollment in the study as well as follow up surveys included phone calls 2–3 weeks after the survey mailing to encourage parents to return the survey and included an offer to complete the survey over the phone. After 3 messages/contacts, a second mailing of the survey was sent to any parent with outstanding surveys. A comprehensive tracking database was employed to record parent dispositions and parent reported reasons for not returning the survey as well as tracking access to the enhanced genomic report via the patient portal.

Descriptive statistics were run on baseline, 3-month post-baseline surveys (usual care arm prior to crossover), and 3-month post-launch surveys (intervention and cross-over). In the case of missing data, when survey measures contained summary scores, a mean score was calculated based on responses provided. Write-in responses for the question regarding who they sought medical information from were categorized by study staff for analysis with the survey data.

Qualitative analyses on interviews were conducted as described in previous publications and relied on review of parent responses to semi-structured interview questions. (Stuckey et al. 2015; Williams et al. 2016) Themes analyzed included satisfaction with the enhanced genomic report, use of the report to communicate with others, technical issues with the EHR report, reasons for using/not using, and suggestions for improvements.

## Results

### Parent Participation and Demographics

After WGS results were disclosed in a traditional genetics visit, all parents from the WGS study (*N* = 84) were randomized and then invited via letter to participate in the GenomeCOMPASS™ study. The participants comprised 42 parental dyads (84 individual parents) and 46 children (Four families had multiple affected children). Four parents were ineligible to participate: one parent died unrelated to the study, and three parents were unavailable for follow-up (one dyad divorced and one parent moved away). Twenty-eight parents did not complete the baseline survey and therefore did not participate in the study. In total, 52 individual parents completed the baseline survey and entered the effectiveness study. (see Fig. 3) Of the 28 in the intervention arm that received the enhanced genomic report, 21 (75%) completed the 3-month post launch survey, 8 participated in short telephone surveys and 1 completed an in-depth interview. Of the 24 parents in the usual care arm, 20 completed the 3-month post baseline survey (83%) and were then sent the enhanced genomic report (asymmetric cross-over). Fifteen (75%) completed the post-report survey, 10 participated in short telephone interviews and 4 completed an in-depth interview.

Participant characteristics at baseline survey are shown in Table 3. Slightly more mothers than fathers completed the baseline survey (58% vs 42%) and most participants were White (96%), married (88%), had at least some college (63%), and were employed (75%). Health literacy and numeracy was high; greater than 60% of parents indicated they were often or always confident filling out medical forms by themselves and greater than 70% indicated they found statistics easy or very easy to understand. Fewer than half of parents reported accessing their child’s medical record through the online MyGeisinger patient portal. Most parents with children in the WGS study received an uninformative result following initial analysis of the whole genome sequences. Of those enrolled, seven parents (14%) received a diagnostic result for their child.

**Table 3:** Participant characteristics

Variant Status	N	%
Causal variant/Diagnostic	7	13.5%
Non-causal variant/Uninformative	45	86.5%
Sex		
Male	21	40.3%
Female	29	55.9%
Missing	2	3.8%
Race/Ethnicity		
White or Caucasian	49	94.3%
Other	2	3.8%
Missing	1	1.9%
Hispanic	2	3.8%
Non-Hispanic	49	94.3%
Missing	1	1.9%
Marital status		
Now married	45	86.5%
Divorced	3	5.8%
Separated	2	3.9%
PCORI	1	1.9%
Missing	1	1.9%
Education		
Some high school (9–12)	4	7.7%
High school graduate or GED	10	19.2%
Post high school training other than college	7	13.5%
Some college	13	25.0%
Bachelor’s degree or equivalent	9	17.3%
Master’s degree	8	15.4%
Doctor or other professional degree	1	1.9%
Employment status		
Working for pay	39	75.0%
other	9	17.0%
Disabled	4	8.0%
Household income		
Less than $15,000	1	1.9%
$15,000 to $29,999	4	7.7%
$30,000 to $44,999	6	11.5%
$45,000 to $59,999	8	15.4%
$60,000 to $89,999	18	34.6%
$90,000 to $149,999	5	9.6%
$150,000 to $199,999	5	9.6%
$200,000 or above	3	5.8%
Missing	2	3.8%
Confidence filling out forms (Health literacy)		
Never	1	1.9%
Occasionally	6	11.5%
Sometimes	9	17.3%
Often	6	11.5%
Always	29	55.8%
Missing	1	1.9%
How find statistics (numeracy)		
Very easy	11	21.6%
Easy	25	49.0%
Hard	15	29.4%
Very hard	0	0.0%
Missing	1	1.9%
Number of times accessed child’s record in patient portal		
None	29	55.8%
1 to 2 times	4	7.7%
3 to 5 times	8	15.4%
6 to 9 times	2	3.8%
10 or more times	7	13.5%
Missing	2	3.8%

### Quantitative Results and Analysis

(All survey items and responses are included in Online Resource 4)

Descriptive statistics were run on baseline, 3-month post-baseline surveys (usual care arm prior to crossover) and at 3-month post report (intervention arm and crossover). In the case of missing data, when survey measures contained summary scores, a mean score was calculated based on responses provided. This statistical method for handling missing information is typical for the scales used (Table 1), however may result in over-estimation of respondent summary score. Clear instructions and pretesting of the survey instrument were used to reduce potential for parents to not answer individual questions. We examined respondent answers to individual questions in each scale as well as summary scores to reduce the impact of using scale scores calculated only on responses provided. Write-in responses for the question regarding from whom they sought medical information were categorized by study staff for analysis. Inferential statistical tests were not conducted due to the very small sample size of parents who opened the report.

#### Baseline Parent Characteristics

General Health of Participant/Child: More than 90% of the parent respondents described their own health as good, very good, or excellent. On a 5 point scale of “Not confident at all” to Completely confident”, all were a little confident or above in their ability to take good care of their health. This was not different between the intervention group and the usual care group. At baseline, 81.1% of parents rated their child’s health as good or better, and parental confidence in their ability to care for their child’s health was rated at 94.3% somewhat confident or above.

Health Information Preferences (DuBenske et al. 2009): The participants in this study are admitted information seekers with overall high information engagement subscale score (mean score = 3) and low information apprehension subscale scores (mean = 0.75). On all items related to information-seeking, greater than 90% affirmed information-gathering behavior. The majority (83%) of parents in both groups indicated that they use the internet to search for information about health or medical topics for their child.

Decision Regret (Brehaut et al. 2003): Most parents had no regret regarding their child’s participation in the WGS clinical research study with 90% agreeing/strongly agreeing it was the right decision, 90% disagree/strongly disagree they regret the choice, and 89% would do it again, regardless of whether or not a diagnostic result was found.

Psychosocial Adjustment to Genetic Information (PAGIS) scale (Read et al. 2005): The seven parents at baseline who received a diagnostic result for their child received this scale and reported certainty subscale score of 4.8 (range 1–5) indicating high baseline certainty in their knowledge about the result.

MICRA Scale (Cella et al. 2002): Most parents (82.4%) were never or rarely upset or sad (74.5% rarely or never) by their child’s result. Nearly all parents (97.9%) reported they never or rarely felt the result made it hard to cope with the child’s diagnosis, however, 60.9% also reported sometimes or often feeling frustrated there were no definite health guidelines for the child.

#### Three-Month Post Baseline and 3-Month Post Report

Due to the low number of parents who opened the report (*n* = 9) and the low number with a diagnostic result (*n* = 7), evaluating for differences between intervention and control at three months or for change between baseline and 3 months post receipt of enhanced report (3-month survey for intervention, 6-month survey for control) was not informative. One measure, the PAGIS scale suggests potential impact of the enhanced report. Four parents with a diagnostic result in the control arm completed this scale at all time points. Their scores indicated high certainty at baseline (mean scale score 4.5), low certainty at 3 months post baseline (no intervention; mean scale score 2.6), and high certainty again at 3 months post-enhanced report (6 months post baseline, 3 months post crossover; mean scale score 4.6).

#### In-Depth and Short Interviews

Per the sequential explanatory design, parents were invited to participate in in-depth qualitative interviews after completing the 3-month post-report survey (3 months post-baseline for intervention and 6 months post baseline for crossover). Due to the low utilization of the report and return of surveys, a short phone call interview was added after the 3-month post report survey to elicit information about access and barriers and then invite the parent to participate in an in-depth interview. Study staff contacted all parents by phone approximately one month after all 3 month post enhanced report surveys had been sent. This resulted in the completion of 8 short interviews and 1 in-depth interview with the intervention group (Fig. 3) plus 10 short interviews and 4 in-depth interviews with the crossover group. As shown in Fig. 3, these short and in-depth interviews were completed both with parents who returned the surveys and those who did not in order to understand the utility and barriers of the enhanced report.

### Report Access

Based on user analytics data from the GenomeCOMPASS™ report in the patient portal, 15 of the 46 available reports (33%) were accessed by parents or providers (9 by parents, 7 by providers). For children with uninformative (results (*N* = 39), 6 reports were accessed by parents and 6 by providers. For children where a diagnostic result was found (*N* = 7), 3 reports were accessed by parents and one by providers. One parent accessed the report 2 times during the study period and the others 1 time each. Of the uninformative result reports, one report was accessed 3 times, two reports were accessed twice and three reports were accessed once.

Interestingly, on the 3-month post-launch surveys, more parents responded they had opened the enhanced report (*N* = 20) than indicated by COMPASS™ utilization metrics (*N* = 15).

### Satisfaction with Enhanced Genomic Report

Survey results revealed that many parents in the intervention arm thought the report was NOT helpful or only “a little bit” helpful at the 3-month survey, in contrast with more parents in the crossover arm who indicated that the report was “quite a bit” or “very” helpful at 3-months post-launch report exposure. Further examination, revealed that the parents who had responded in the intervention arm had all received an uninformative result for their child, while 4 of the parents responding in the crossover arm had received a diagnostic result. Parents who received an uninformative result in the crossover arm did not answer the survey questions about the enhanced genomic report. Follow up structured interviews with a subset of parents who received an uninformative result confirmed that parents liked the report structure, language, and delivery method, but felt that there was little value in having a report about a negative result. One mother put it best, *“The report I want is the one that helps my boys.”*

### Parental Utilization of Report

Parents who received a diagnostic result and accessed the enhanced genomic report expressed their satisfaction with the report content and accessibility. Two mothers who both received diagnostic results for their children revealed, through qualitative interviews responses, the value and impact this patient-centered enhanced genomic report had for them and how the report improved the interpretation of their child’s complex results. They described using the report to understand recommended care. They indicated that the report enhanced communication and shared decision-making and empowered them to participate in coordinating their child’s healthcare. Both mothers utilized the report as a printout, sending it via email, and/or accessing it through a phone/tablet when meeting with other physicians inside and outside of Geisinger, when meeting with new teachers and other specialists, and sharing it with “*anyone who is interested in her*.”

*“She had a new speech therapist and a new occupational therapist, and …T* *hey were both really grateful to have the information with her syndrome, as far as ways to treat her and help her* *, and then … she was in the hospital and … the hospitalist … had gone over everything, and used the information in the packet, so I thought that was pretty great.”* (#1537)

*“We were actually at the National Institute of Health a few weeks ago ...and they got all of her records from Geisinger* *except for anything with her mutation on it or saying anything about it* *. So I was actually able to print [the report] out for them and give it to them while we were down there so… It's my like go to place. You know, if I need something quick I know where it's at, at least.”* (#1541)

Access outside of the Geisinger system was critical for this mother, and the report format and prognostic table helped guide her daughter’s care in directions that would not have been possible without the report. Importantly, this sentiment was also expressed by the NIH providers through personal contact (unsolicited) with the study principal investigator as well as the parent.

### Impact on Communication and Information Asymmetry

Though limited in number, the parents with children who received a diagnosis, found the enhanced genomic report aided in communication about the genetic finding and the rare condition. One mother described the value to her as a parent in terms of being able to communicate with medical professionals because *“[showing the report is] easier than me trying to explain it to medical professionals when I might not be able to explain it to the degree that they want me to or* *sometimes they don't think I know what I'm talking about.* *”* The report has helped her *“in understanding and then* *being able to explain to others what's wrong with my daughter* *…* *I felt like it evened the table… I don't feel so overwhelmed* *when I'm discussing it with doctors or other medical professionals because* *I feel like I'm informed enough to know what I'm talking about* *.”* The comment about not feeling overwhelmed because it “evened the table” is particularly salient as one of the anticipated benefits of the report articulated in the proposal was reduction of information asymmetry. Most importantly, when asked to sum up the value of the enhanced report to her this parent explained, *“it* *makes me feel like the best advocate that I can be for my daughter* *…whether it's medical treatment or it’s education, or therapy, I can help guide the direction it goes in because* *I'm learning and I'm using this tool that I have to understand what she needs* *.”* (#1537)

### Technical Challenges

Technical difficulties with the delivery of the GenomeCOMPASS™ report were not recognized until participating parents were contacted about use of the tool. Although beta testing of the tool was completed and all navigation challenges addressed, the actual messaging in the patient portal that announced the availability of the GenomeCOMPASS™ report had not been previewed. The generic COMPASS™ has previously been used almost exclusively for deployment of surveys to clinical care. In this launch, the generic language (“a survey is available”) in the COMPASS message did not alert parents to the fact that their child’s genome results were now available. Once this was reported, the team developed an instruction sheet to outline the step-by-step process to access the enhanced report and sent it to all parents as reports were launched. A letter was sent to parents explaining that they would be notified via a MyGeisinger (patient portal) message that a survey was available and to follow the instructions on the accompanying handout in order to access their child’s genome results report. The letter included the contact information of study personnel for assistance if parents did not receive a message or had difficulty accessing the enhanced genomic report. Study personnel received no requests for assistance after sending this information.

Follow up interviews revealed that despite this additional information, parents were still confused about accessing the report. Parents reported that the tool name, COMPASS™, had no meaning for them. Even though “COMPASS” is mentioned in the email and is available as a link in their portal menu, they expressed varying ideas of what they thought it was– none of which were related to a test report. While parents reported this confusion in the interviews, no parents had reached out to project staff for assistance after receiving the instructions and notice about the enhanced report.

Parents also reported in follow up interviews that the standard subject line of the email message stating “a survey is available” was problematic because it did not differentiate the report from the other surveys they receive using the same subject line. Parents who do use the patient portal further commented that they receive so many inbox messages about surveys, lab results, and appointments from their own and their child’s accounts that they often do not open the message unless the alert/subject line differentiates the message subject from the other messages.

Finally, despite most parents indicating use of the patient portal, MyGeisinger, during the results session of the WGS study, fewer than half reported accessing their child’s medical record on the baseline survey. Follow-up interviews clarified that some parents were unaware that they had to officially “connect” to their child’s medical record in order to access that record; parents not connected to the child’s MyGeisinger account, therefore, did not receive the message that the enhanced genomic report was available. Again, while parents reported this on follow-up interviews, no parents reached out to project staff after receiving the instructions and notice about the enhanced genomic report availability.

## Discussion

We hypothesized that an enhanced genomic result report (a report created with engagement of end-user parents and providers and available electronically through the medical record system and patient portal) would improve communication and engagement while reducing the information asymmetry that exists between patients, primary care providers and genetics providers, particularly in the context of rare genetic disease. The primary outcomes included measures of the actual use of and satisfaction with the enhanced genomic report. As listed in Table 1, primary and secondary outcomes were obtained using qualitative and quantitative measures. The intentional use of mixed-methods design was critical to evaluation and understanding of study results, which were hampered by limited sample size and unanticipated low number of parents who received a diagnostic result. Inclusion of qualitative assessment within the study design illuminated important issues around access and satisfaction and revealed potential lack of value in a report to parents when their child’s result was negative. Throughout this study, our parent participants confirmed the challenges of interpreting and communicating genetic results, a point that is repeatedly made in the literature. (Greco and Mahon 2011; Condit 2010; Suther and Goodson 2003) Parents also anticipated utility of the report for settings outside of health (i.e. school, therapy, etc.) during the design phase. Parents reported that these non-health professionals found the report valuable and unsolicited reviews from external health providers also reiterated the effectiveness of the enhanced report to improve communication, direct care management, and encourage confidence in understanding the rare condition and reduce information asymmetry. Parents who received a diagnostic result reported through interviews that the enhanced genomic report as implemented in the study did, in fact, empower them, reduce information asymmetry (per a parent’s own words), and enhance communication between with many different providers and caregivers inside and outside of the healthcare system. The fact that parents used the report this way and that other providers also expressed value in the enhanced report further underscores the importance of patient and end-user engagement in this type of research.

Based on the developmental work (Stuckey et al. 2015), the study team hypothesized that an enhanced genomic report would be valuable to parents, whether or not a diagnosis had been found. The team carefully randomized for diagnosis before launch of the enhanced genome report, recognizing that there could be differences in use of the report. In the developmental work, parents had also indicated that a report of negative findings would be useful, particularly if it contained links to vetted resources. As the team analyzed the results of the survey in the intervention and cross-over arms, it became apparent that parents whose child had a diagnosis opened the report and those with an uninformative result did not access the report. The survey data also indicated that parents reported opening the Genome COMPASS™ report more often than the analytics supported. We surmise that parents may have confused the summary letter following their results visit for the enhanced genomic report.

When we interviewed parents who received an uninformative result, they confirmed they did not find the enhanced genomic report useful; stating they found little reason to open the GenomeCOMPASS™ report as there were no findings to learn about. A similar disappointment has been seen in other studies where parents had expected to find a diagnostic result from WGS (Cacioppo et al. 2016; Sapp et al. 2014). This is also an example of the oft seen difference found in research between what is described as important in a developmental phase (even with significant end-user engagement) and what becomes apparent once the end-product is implemented (Dearing and Kreuter 2010; Lerman et al. 1994). However, for those who accessed the enhanced genomic report, they reported satisfaction with the content and delivery. This indicated that access and satisfaction with the GenomeCOMPASS™ report may be tied to whether a diagnostic result is found, rather than dissatisfaction with the report language, structure, content, or delivery.

### Future Directions

While promising and innovative approaches to patient-controlled genomic data sharing such as *GenomeConnect*, *My46*, or *MyGene2* are emerging, the number of engaged patients is in the low thousands and **none of these approaches is integrated with an EHR or the healthcare delivery system.** The data from this study suggest that an enhanced genomic report developed in conjunction with feedback from end-users, implemented in a healthcare system, and delivered through the EHR could be useful for reporting genomic sequencing results for children with Intellectual Disability and Autism Spectrum Disorder.

The general COMPASS™ tool has been integrated throughout Geisinger, and the Genome COMPASS™ report has been approved for use for the return of all clinical and research genomic results. Pharmacogenomic COMPASS™ reports are also under development using a similar patient and provider (Physician and Pharmacist) engagement process to develop report language and content prior to implementation. Plans are in place to make the GenomeCOMPASS™ tool compatible with SMART genomics and Fast Healthcare Interoperable Resources, *FHIR*, (FHIR-HL7wiki n.d.)which should improve generalizability to other EHR systems by removing compatibility barriers. (Alterovitz et al. 2015).

### Limitations

The results of the effectiveness study are limited in that we had fewer parents accessing the enhanced report; in part, due to having fewer diagnostic findings to report than anticipated. These limitations, however, are mitigated by the mixed-methods design which included rich interview data that helped to interpret the survey and place access and satisfaction results in context. Interview data indicated the low uptake was due to a lack of utility when results were negative rather than a deficit in the enhanced report itself, while those who received a diagnostic result reported utilizing their GenomeCOMPASS™ report exactly as designed. Other limitations included unexpected technical issues that interfered with the access to the report by families, such as parents not realizing they needed to have proxy access to their child’s record to see the report and the stock COMPASS™ message language not distinguishing the genomic results report from other surveys patients receive through the tool. These barriers were reported despite additional information and instruction about COMPASS™ and the report being sent via mail to all parents. Additional ways to address these technical issues are being explored for further implementation and study. Finally, the usual care results return process was very thorough and perhaps contributed to a ceiling effect on satisfaction, understanding, and confidence reported on the baseline surveys. Parents felt highly informed and confident in dealing with their child’s condition, and the summary letter of the results visit typically sent after each genetics visit may have been confused with the enhanced report; resulting in more parents reporting accessing the enhanced report and limiting the ability to detect change using the survey measures.

### Recommendations for Future Research

Although we believe that the enhanced genomic report should perform well across all demographic groups, given the universal impact of rare genetic disorders in these groups, further deployment and evaluation of effectiveness in a broad range of patient end-users is desirable. A more robust assessment could provide insight into how the report’s utility may be affected by factors such as race/ethnicity; socioeconomic status; educational level and health literacy; and membership in an underserved and vulnerable population. Making the report available in different languages is important, although does not define a research agenda in and of itself as long as end-users are engaged to confirm the fidelity of the report. The focus of future research should be to gather additional effectiveness through hybrid designs with dissemination and implementation (hybrid effectiveness-implementation studies) in different settings and with adaptation for diverse indications.

### Implications for Genetic Counseling Practice

Genetic counselors are expected to provide meaningful information to patients pertaining to a variety of genetic testing results and settings in a way that conveys accurate genetics results content, vetted patient resources and optimal management recommendations. This becomes more challenging in the context of whole exome or whole genome sequence results when primary and secondary genomic results may be found. Templated genomic test reports that pull in the applicable genomic content, with versioning options as genomic sequences are reanalyzed or results are re-interpreted, and provide external links to patient resources can be a supportive EHR tool that provides patient and provider access to the information needed to understand and manage a variety of genetics results scenarios including pharmacogenomics, ultra-rare Mendelian disease and secondary findings such as the ACMG list (Green et al. 2013; Kalia et al. 2016). This report, may offer an alternative to the traditional summary letter used by many genetics professionals following patient visits.

## Conclusion

Results of this study suggest that a patient-centered report developed by parents to empower and facilitate communication works as designed: Parents for whom the report was most relevant were highly satisfied with the report, and reported that they felt more confident and better able to advocate for their child (regardless of already high levels of confidence and advocacy) and that they had shared the report with multiple providers and caregivers. Unsolicited communication from external providers confirmed the value of the enhanced report to providers in making the information accessible, thus facilitating the most effective care for the child. Further, this tool, which can be adapted in many EHR settings, offers the opportunity to support patients, their families and healthcare providers using the EHR as the vehicle to disseminate accurate genetic information, provide support real-time management support and to connect with appropriate resources regarding rare conditions. This study provides an important step in the process to make genomic information available and relevant for patients and providers.

## Electronic Supplementary Material

ESM 1 (PDF 653 kb)

ESM 2 (PDF 484 kb)

ESM 3 (PDF 212 kb)

ESM 4 (PDF 795 kb)
