# Supplementary material for: Impact of a Patient-Facing Enhanced Genomic Results Report to Improve Understanding, Engagement, and Communication
Source: J Genet Couns. 2017 Dec 4;27(2):358–69. doi: 10.1007/s10897-017-0176-6 (PMC5859697; doi:10.1007/s10897-017-0176-6)
Supplement: Supplementary file 1 — (PDF 653 kb) [file 10897_2017_176_MOESM1_ESM.pdf]

BASELINE QUESTIONNAIRE
To be completed by both groups at beginning of trial

Study ID#___________
Date:______________

Section 1. General Health

Please answer the first question about yourself. The questions that follow will be about your child who participated in the whole genome sequencing study.

1. In general, how would you describe **your** health?

   1. Excellent
   2. Very good
   3. Good
   4. Fair
   5. Poor

2. Overall, how confident are you about your ability to take good care of **your** health?

   1. Completely confident
   2. Very confident
   3. Somewhat confident
   4. A little confident
   5. Not confident at all

Please answer the next questions about your child's health:

5. In general, how would you describe **your child's** health?

   1. Excellent
   2. Very good
   3. Good
   4. Fair
   5. Poor

6. Overall, how confident are you about your ability to take good care of **your child's** health?

   1. Completely confident
   2. Very confident
   3. Somewhat confident
   4. A little confident
   5. Not confident at all

Section 2. Health Information Preferences

The following questions ask about how you like to gather information about health concerns.

1. When you are dealing with health concerns for yourself or your child, how do you like to handle things? Please answer how true each of the following statements is for you:

	Not at all true (0)	A little bit (1)	Somewhat true (2)	Quite a bit (3)	Very much true (4)
I like to gather as much information as I can before making a decision	0	1	2	3	4
I like to review information multiple times before making a decision	0	1	2	3	4
After I've made a decision, I continue to look for related information	0	1	2	3	4
I like to make decisions quickly	0	1	2	3	4
I have difficulty making sense of information from multiple sources	0	1	2	3	4
I fear that I might find out something I don't want to know	0	1	2	3	4
I feel overwhelmed by the amount of information available	0	1	2	3	4
I think it's the doctor's job to deal with information, not mine	0	1	2	3	4

2. Do you ask family members or friends for information or advice on health topics?
   1. Yes
   2. No

3. Who do you ask for this information? (fill in)

Section 3. Internet use and information seeking

1. Have you ever looked for information about health or medical topics for your child from any source?
   1. Yes
   2. No (Skip to question 5)

2. The most recent time you looked for information about health or medical topics for your child where did you go first? Please circle your top choice.
   1. Books
   2. Brochures, pamphlets, etc.
   3. Cancer organization
   4. Family
   5. Friend/Co-worker
   6. Doctor or health care provider
   7. Internet
   8. Library
   9. Magazines
   10. Newspapers
   11. Telephone information number
   12. Complementary, alternative, or unconventional practitioner
   13. Genetic test report

3. Did you look or go anywhere else that time?
   1. Yes (If Yes, where____)
   2. No

4. Based on the results of your most recent search for information about health or medical topics regarding your child, how much do you agree or disagree with each of the following statements?

	Strongly Agree (1)	Somewhat Agree (2)	Somewhat Disagree (3)	Strongly Disagree (4)
It took a lot of effort to get the information you needed	1	2	3	4
You felt frustrated during your search for the information	1	2	3	4
You were concerned about the quality of the information	1	2	3	4
The information you found was hard to understand	1	2	3	4

5. Overall, how confident are you that you can get advice or information about health or medical topics if you needed it?

1. Completely confident
2. Very confident
3. Somewhat confident
4. A little confident
5. Not at all confident

Section 4. Provider Communication

1. In general, how often do you do each of the following:

	Always (1)	Usually (2)	Sometimes (3)	Never (4)
Take with you to your child's doctor visits a list of questions or concerns you want to cover	1	2	3	4
Take a list of all of your child's prescribed medicines to his/her doctor visits	1	2	3	4
Ask your child's doctor to explain a test, treatment, or procedure to you in detail	1	2	3	4
Read information about a new prescription, such as side effects and precautions	1	2	3	4
Do your own research on a health or medical topic after seeing your child's doctor	1	2	3	4
Take with you to your child's doctor visit any kind of health information you have found	1	2	3	4

2. Have you ever talked to your child's doctor, nurse, or other health care provider about any kind of health information you have gotten from the internet?

1. Yes
2. No (Skip to question 5)

3. When you talked with your child’s health care provider, how interested were they in hearing about the information you found online? Were they….

1. Very interested
2. Somewhat interested
3. A little interested
4. Not at all interested

4. The following questions are about your communication with your child’s doctors, nurses, or other health professionals you saw during the past 12 months.

How often did they do each of the following:	Always (1)	Usually (2)	Sometimes (3)	Never (4)
Give you the chance to ask all the health-related questions you had?	1	2	3	4
Give the attention you needed to your feelings and emotions?	1	2	3	4
Involve you in decisions about your child’s health care as much as you wanted?	1	2	3	4
Make sure you understood the things you needed to do to take care of your child’s health?	1	2	3	4
Explain things in a way you could understand?	1	2	3	4
Spend enough time with you?	1	2	3	4
Help you deal with feelings of uncertainty about your child’s health or health care?	1	2	3	4

5. Overall, how would you rate the quality of health care your child received in the past 12months?

1. Excellent
2. Very good
3. Good
4. Fair
5. Poor

6. In the past 12 months, how often did you feel you could rely on your doctors, nurses, or other health care professionals to take care of your child's health care needs?

1. Always
2. Usually
3. Sometimes
4. Never

Section 5. Participating in Whole Genome Sequencing Research Study

Sometimes people feel differently about genetic testing decisions after they receive results. When you think about having gone through whole genome testing for your child and the results you received, how much would YOU now agree or disagree with each of the following statements?

	Strongly Agree (1)	Agree (2)	Neither Agree nor Disagree (3)	Disagree (4)	Strongly Disagree (5)
It was the right decision	1	2	3	4	5
I regret the choice that was made	1	2	3	4	5
I would go for the same choice if I had to do it over again	1	2	3	4	5
The choice did me a lot of harm	1	2	3	4	5
The decision was a wise one	1	2	3	4	5

(Continue to next page)

Section 6. Response to whole genome sequencing results

(Note: For those with positive results)

	Strongly Agree (1)	Agree (2)	Neither Agree nor Disagree (3)	Disagree (4)	Strongly Disagree (5)
I understand how my child came to have this gene	1	2	3	4	5
I understand the health risks my relatives face because of my child having this gene.	1	2	3	4	5
I feel certain that I understand the meaning of my child having this gene	1	2	3	4	5
I understand the chances I have of passing this gene along to other children	1	2	3	4	5
I feel that I can explain to other people what my child having this gene means	1	2	3	4	5

Section 7. Response to whole genome sequencing results

The statements below reflect responses some people have after learning a child's genetic test result. Please respond how much each of these statement represents how you have felt ***in the past week***.

**In the past week,** how often have you been…	Never	Rarely	Sometimes	Often
Feeling upset about your child's test results	1	2	3	4
Feeling sad about your child's test results	1	2	3	4
Feeling anxious or nervous about your child's results	1	2	3	4
Feeling guilty about your child's results	1	2	3	4
**In the past week,** how often	Never	Rarely	Sometimes	Often

have you been…				
Feeling relieved about your child's test results	1	2	3	4
Feeling happy about your child's results	1	2	3	4
Feeling loss of control	1	2	3	4
Having problems enjoying your life because of your child's results	1	2	3	4
Worrying about your child's risk of becoming sick or ill	1	2	3	4
Being uncertain about what your child's results mean about your child's future health	1	2	3	4
Being uncertain about what your child's results mean for any other children and/or your family's future health	1	2	3	4
Having difficulty making decisions about health screening or disease prevention for your child	1	2	3	4
Understanding clearly your child's choices for health screening or disease prevention	1	2	3	4
Feeling frustrated that there are no definite health guidelines for your child	1	2	3	4
Thinking about your child's result has affected your work or family life	1	2	3	4
Feeling concerned about how your child's results will affect your insurance status	1	2	3	4
Having difficulty talking about your child's results with family members	1	2	3	4
Feeling that your family has been supportive during the process of learning the genetic result	1	2	3	4
Feeling satisfied with family communication about your child's genetic results	1	2	3	4
**In the past week,** how often have you been…	Never	Rarely	Sometimes	Often

Worrying that your child's results have brought about conflict within your family	1	2	3	4
Feeling regret about getting the test result	1	2	3	4
Feeling that the genetic test result has made it <u>harder</u> to cope with my child's diagnosis	1	2	3	4
Feeling that the genetic test result has made it <u>easier</u> to cope with my child's diagnosis	1	2	3	4

Section 8. Questions about the report you received on your child's genetic testing

1. Did you read the letter describing your child's whole genome sequencing results?
    1. Yes
    2. No (Skip remaining questions)

2. How helpful did you find the various parts of the **genetic testing** report you received?

	Not helpful (0)	A little bit helpful(1)	Somewhat helpful (2)	Quite a bit helpful (3)	Very helpful (4)
The summary	0	1	2	3	4
The detailed explanation	0	1	2	3	4
The care instructions	0	1	2	3	4

3. Did you review the genetic testing report you received with your provider?
    1. Yes, in person
    2. Yes, over the phone
    3. No (skip question 4 and 5)

4. Did reviewing the genetic testing report with your provider help improve your understanding of your child's health condition?
    n/a No, I already understood everything I needed (n/a)
    0. Didn't help
    1. Helped a little
    2. Helped some
    3. Helped a fair amount
    4. Helped a great deal

5. Do you have any comments you want to share about the report? (final open ended question)

Section 9. Comfort with how health information is described

1. How confident are you filling out forms by yourself?
   0. Never
   1. Occasionally
   2. Sometimes
   3. Often
   4. Always

2. In general, how easy or hard do you find it to understand medical statistics?
   1. Very easy
   2. Easy
   3. Hard
   4. Very hard

3. How much do you agree or disagree with the following statement? *In general, I depend on numbers and statistics to help me make decisions about my child's health?*
   1. Strongly agree
   2. Somewhat agree
   3. Somewhat disagree
   4. Strongly disagree

4. Which of the following numbers represents the biggest risk of getting a disease?
   1. 1 in 100
   2. 1 in 1000
   3. 1 in 10

5. People can talk about the chance of something happening using either words, like "*It rarely happens*" or numbers, like "*There's a 5% chance*." When people tell you the chance of something happening, do you prefer they use words or numbers?
   1. Generally prefer words
   2. Generally prefer numbers
   3. No preference

6. How many times did you access your child's health information online through the MyGeisinger website or app in the last 12 months?
   1. None

2. 1 to 2 times
3. 3 to 5 times
4. 6 to 9 times
5. 10 or more times

Section 10. Demographics

Please complete the following questions answering for yourself rather than for your child unless the question specifically asks you for an answer about your child. All information that is requested is standard in research studies so that the population as a whole can be described. Your answers will confidential and tied to a study number and not used to identify you personally.

1. What is your age? _______________Years

2. Are you Male or Female? (Please circle)
   1. Male
   2. Female

3. Which one or more of the following would you say is your race? Please check all that apply.
   1. _____White or Caucasian
   2. _____Black or African American
   3. _____American Indian or Alaska Native
   4. _____Asian
   5. _____Pacific Islander
   6. _____Other
   7. _____Don't know

4. Are you Hispanic or Latino/a?
   1. Yes
   2. No
   3. Don't know

6. What is your current marital status?
   1. Now married (skip question 7)
   2. Widowed
   3. Divorced
   4. Separated
   5. Never married

7. Are you currently living with a boyfriend/girlfriend or partner?
   1. Yes
   2. No

8. What is the highest grade or year or school you completed? Mark only ONE response.
   1. Never attended school
   2. Grade school (grades 1-8)

3. Some high school (grades 9-12)
4. High school graduate or GED
5. Post high school training other than college (vocational, technical, or other types of training)
6. Some college
7. Bachelor's degree or equivalent
8. Master's degree (MS, MBA, MFA, etc)
9. Doctoral or other professional degree (PhD, MD, JD or other)

9. What is your current work situation? Mark all that apply.

1. ___Working
2. ___Only temporarily laid off, sick leave or maternity leave
3. ___Looking for work, unemployed
4. ___Retired
5. ___Disabled, permanently or temporarily
6. ___Homemaker
7. ___Student
8. ___Other

10. What is your household's total combined income during the past 12 months? (This includes money from pensions, social security payments, jobs, net income from business, farm or rent, dividends, interest and any other income received by family members who are 15 years of age or older.)

1. Less than $15,000
2. $15,000 to $29,999
3. $30,000 to $44,999
4. $45,000 to $59,999
5. $60,000 to $89,999
6. $90,000 to $149,999
7. $150,000 to $199,999
8. $200,000 or above

THANK YOU!!!!
