# Supplementary material for: Impact of a Patient-Facing Enhanced Genomic Results Report to Improve Understanding, Engagement, and Communication
Source: J Genet Couns. 2017 Dec 4;27(2):358–69. doi: 10.1007/s10897-017-0176-6 (PMC5859697; doi:10.1007/s10897-017-0176-6)
Supplement: Supplementary file 2 — (PDF 484 kb) [file 10897_2017_176_MOESM2_ESM.pdf]

**3 Month** Study ID#___________

*To be completed by both groups 3 months after start* Date:______________

## Section 1. General Health

Please answer the first question about yourself. The questions that follow will be about your child who participated in the whole genome sequencing study.

1. In general, how would you describe **your** health?
   1. Excellent
   2. Very good
   3. Good
   4. Fair
   5. Poor

2. Overall, how confident are you about your ability to take good care of **your** health?
   1. Completely confident
   2. Very confident
   3. Somewhat confident
   4. A little confident
   5. Not confident at all

**Please answer the next questions about your child's health:**

5. In general, how would you describe **your child's** health?
   1. Excellent
   2. Very good
   3. Good
   4. Fair
   5. Poor

6. Overall, how confident are you about your ability to take good care of **your child's** health?
   1. Completely confident
   2. Very confident
   3. Somewhat confident
   4. A little confident
   5. Not confident at all

## Section 2. Health Information Preferences

**The following questions ask about how you like to gather information about health concerns.**

1. When you are dealing with health concerns for yourself or your child, how do you like to handle things? Please answer how true each of the following statements is for you:

| | Not at all true (0) | A little bit (1) | Somewhat true (2) | Quite a bit (3) | Very much true (4) |
|---|---|---|---|---|---|
| I like to gather as much information as I can before making a decision | 0 | 1 | 2 | 3 | 4 |
| I like to review information multiple times before making a decision | 0 | 1 | 2 | 3 | 4 |
| After I've made a decision, I continue to look for related information | 0 | 1 | 2 | 3 | 4 |
| I like to make decisions quickly | 0 | 1 | 2 | 3 | 4 |
| I have difficulty making sense of information from multiple sources | 0 | 1 | 2 | 3 | 4 |
| I fear that I might find out something I don't want to know | 0 | 1 | 2 | 3 | 4 |
| I feel overwhelmed by the amount of information available | 0 | 1 | 2 | 3 | 4 |
| I think it's the doctor's job to deal with information, not mine | 0 | 1 | 2 | 3 | 4 |

2. Do you ask family members or friends for information or advice on health topics?
   1 Yes
   2 No

3. Who do you ask for this information? (fill in)

____________________
____________________
____________________
____________________
____________________

## Section 3. Internet use and information seeking

1. Have you ever looked for information about health or medical topics for your child from any source?
    1. Yes
    2. No (Skip to question 5 )

2. The most recent time you looked for information about health or medical topics for your child where did you go first? Please circle your top choice.
    1. Books
    2. Brochures, pamphlets, etc.
    3. Cancer organization
    4. Family
    5. Friend/Co-worker
    6. Doctor or health care provider
    7. Internet
    8. Library
    9. Magazines
    10. Newspapers
    11. Telephone information number
    12. Complementary, alternative, or unconventional practitioner
    13. Genetic test report

3. Did you look or go anywhere else that time?
    1. Yes (If Yes, where_______________________________________________________)
    2. No

4. Based on the results of your most recent search for information about health or medical topics regarding your child, how much do you agree or disagree with each of the following statements?

| | Strongly Agree (1) | Somewhat Agree (2) | Somewhat Disagree (3) | Strongly Disagree (4) |
|---|---|---|---|---|
| It took a lot of effort to get the information you needed | 1 | 2 | 3 | 4 |
| You felt frustrated during your search for the information | 1 | 2 | 3 | 4 |
| You were concerned about the quality of the information | 1 | 2 | 3 | 4 |
| The information you found was hard to understand | 1 | 2 | 3 | 4 |

5. Overall, how confident are you that you can get advice or information about health or medical topics if you needed it?
1. Completely confident
2. Very confident
3. Somewhat confident
4. A little confident
5. Not at all confident

## Section 4. Provider Communication

1. In general, how often do you do each of the following:

| | Always (1) | Usually (2) | Sometimes (3) | Never (4) |
|---|---|---|---|---|
| Take with you to your child's doctor visits a list of questions or concerns you want to cover | 1 | 2 | 3 | 4 |
| Take a list of all of your child's prescribed medicines to his/her doctor visits | 1 | 2 | 3 | 4 |
| Ask your child's doctor to explain a test, treatment, or procedure to you in detail | 1 | 2 | 3 | 4 |
| Read information about a new prescription, such as side effects and precautions | 1 | 2 | 3 | 4 |
| Do your own research on a health or medical topic after seeing your child's doctor | 1 | 2 | 3 | 4 |
| Take with you to your child's doctor visit any kind of health information you have found | 1 | 2 | 3 | 4 |

2. Have you ever talked to your child's doctor, nurse, or other health care provider about any kind of health information you have gotten from the internet?
1. Yes
2. No (Skip to question 6)

3. When you talked with your child's health care provider, how interested were they in hearing about the information you found online? Were they….
1. Very interested

2. Somewhat interested
3. A little interested
4. Not at all interested

5. The following questions are about your communication with your child's doctors, nurses, or other health professionals you saw during the past 3 months.

| How often did they do each of the following: | Always (1) | Usually (2) | Sometimes (3) | Never (4) |
|---|---|---|---|---|
| Give you the chance to ask all the health-related questions you had? | 1 | 2 | 3 | 4 |
| Give the attention you needed to your feelings and emotions? | 1 | 2 | 3 | 4 |
| Involve you in decisions about your child's health care as much as you wanted? | 1 | 2 | 3 | 4 |
| Make sure you understood the things you needed to do to take care of your child's health? | 1 | 2 | 3 | 4 |
| Explain things in a way you could understand? | 1 | 2 | 3 | 4 |
| Spend enough time with you? | 1 | 2 | 3 | 4 |
| Help you deal with feelings of uncertainty about your child's health or health care? | 1 | 2 | 3 | 4 |

6. Overall, how would you rate the quality of health care your child received in the past 3 months?
1. Excellent
2. Very good
3. Good
4. Fair
5. Poor

7. In the past 3 months, how often did you feel you could rely on your doctors, nurses, or other health care professionals to take care of your child's health care needs?
1. Always
2. Usually

3. Sometimes
4. Never

## Section 5. Participating in Whole Genome Sequencing Research Study

Sometimes people feel differently about genetic testing decisions after they receive results. When you think about having gone through whole genome testing for your child and the results you received, how much would YOU now agree or disagree with each of the following statements?

| | Strongly Agree (1) | Agree (2) | Neither Agree nor Disagree (3) | Disagree (4) | Strongly Disagree (5) |
|---|---|---|---|---|---|
| It was the right decision | 1 | 2 | 3 | 4 | 5 |
| I regret the choice that was made | 1 | 2 | 3 | 4 | 5 |
| I would go for the same choice if I had to do it over again | 1 | 2 | 3 | 4 | 5 |
| The choice did me a lot of harm | 1 | 2 | 3 | 4 | 5 |
| I feel that I can explain to other people what having this gene means | 1 | 2 | 3 | 4 | 5 |
| The decision was a wise one | 1 | 2 | 3 | 4 | 5 |

(Continue to next page)

## Section 6. Response to whole genome sequencing results

(Note: For those with positive results)

| | Strongly Disagree (1) | Disagree (2) | Neutral (3) | Agree (4) | Strongly Agree (5) |
|---|---|---|---|---|---|
| I understand how my child came to have this gene | 1 | 2 | 3 | 4 | 5 |
| I understand the health risks my relatives face because of my child having this gene. | 1 | 2 | 3 | 4 | 5 |
| I feel certain that I understand the meaning of my child having this gene | 1 | 2 | 3 | 4 | 5 |
| I understand the chances I have of passing this gene along to other children | 1 | 2 | 3 | 4 | 5 |
| I feel that I can explain to other people what my child having this gene means | 1 | 2 | 3 | 4 | 5 |

## Section7. Response to whole genome sequencing results (For all)

The statements below reflect responses some people have after learning a child's genetic test result. Please respond how much each of these statement represents how you have felt ***in the past week***.

| **In the past week,** how often have you been… | Never | Rarely | Sometimes | Often |
|---|---|---|---|---|
| Feeling upset about your child's test results | 1 | 2 | 3 | 4 |
| Feeling sad about your child's test results | 1 | 2 | 3 | 4 |
| Feeling anxious or nervous about your child's results | 1 | 2 | 3 | 4 |
| Feeling guilty about your child's results | 1 | 2 | 3 | 4 |
| Feeling relieved about your child's test results | 1 | 2 | 3 | 4 |
| Feeling happy about your child's results | 1 | 2 | 3 | 4 |
| Feeling loss of control | 1 | 2 | 3 | 4 |
| Having problems enjoying your life because of your child's results | 1 | 2 | 3 | 4 |
| Worrying about your child's risk of becoming sick or ill | 1 | 2 | 3 | 4 |
| Being uncertain about what your child's results mean about your child's future health | 1 | 2 | 3 | 4 |
| Being uncertain about what your child's results mean for any other children and/or your family's future health | 1 | 2 | 3 | 4 |
| Having difficulty making decisions about health screening or disease prevention for your child | 1 | 2 | 3 | 4 |
| Understanding clearly your child's choices for health screening or disease prevention | 1 | 2 | 3 | 4 |
| Feeling frustrated that there are no definite health guidelines for your child | 1 | 2 | 3 | 4 |

| | | | | |
|---|---|---|---|---|
| Thinking about your child's result has affected your work or family life | 1 | 2 | 3 | 4 |
| Feeling concerned about how your child's results will affect your insurance status | 1 | 2 | 3 | 4 |
| Having difficulty talking about your child's results with family members | 1 | 2 | 3 | 4 |
| Feeling that your family has been supportive during the process of learning the genetic result | 1 | 2 | 3 | 4 |
| Feeling satisfied with family communication about your child's genetic results | 1 | 2 | 3 | 4 |
| Worrying that your child's results have brought about conflict within your family | 1 | 2 | 3 | 4 |
| Feeling regret about getting the test result | 1 | 2 | 3 | 4 |
| Feeling that the genetic test result has made it <u>harder</u> to cope with my child's diagnosis | 1 | 2 | 3 | 4 |
| Feeling that the genetic test result has made it <u>easier</u> to cope with my child's diagnosis | 1 | 2 | 3 | 4 |

## Section 8. Questions about the report you received on your child's genetic testing

(Baseline-reference results section), (3mo/6mo-reference report/enhanced report)

1. Did you read the {report]/ [letter] describing your child's whole genome sequencing results? (Not at baseline)
    a. Yes
    b. No (Skip remaining questions)

2. How helpful did you find the various parts of the **genetic testing** report you received?

| | Not helpful (0) | A little bit helpful(1) | Somewhat helpful (2) | Quite a bit helpful (3) | Very helpful (4) |
|---|---|---|---|---|---|
| The summary | 0 | 1 | 2 | 3 | 4 |
| The detailed explanation | 0 | 1 | 2 | 3 | 4 |
| The care instructions | 0 | 1 | 2 | 3 | 4 |

3. Did you review the genetic testing report you received with your provider?
    a. Yes, in person
    b. Yes, over the phone
    c. No (skip question 4 and 5)

4. Did reviewing the genetic testing report with your provider help improve your understanding of your child's health condition?
    d. No, I already understood everything I needed (n/a)
    e. Didn't help (0)
    f. Helped a little (1)
    g. Helped some (2)
    h. Helped a fair amount (3)
    i. Helped a great deal (4)

5. Do you have any comments you want to share about the report? (final open ended question)

________________________________________________________________________________

________________________________________________________________________________

________________________________________________________________________________

**THANK YOU!!!!**
