# Supplementary material for: Impact of a Patient-Facing Enhanced Genomic Results Report to Improve Understanding, Engagement, and Communication
Source: J Genet Couns. 2017 Dec 4;27(2):358–69. doi: 10.1007/s10897-017-0176-6 (PMC5859697; doi:10.1007/s10897-017-0176-6)
Supplement: Supplementary file 3 — (PDF 212 kb) [file 10897_2017_176_MOESM3_ESM.pdf]

Participants: Semi-Structured Interview Guide POST Patient-Facing Genome Report

Introduction:

Thank you for agreeing to take part in this study. As a reminder, your participation in this study is voluntary. You have been asked to participate in this interview because you are part of the whole genome sequencing study and received a specially-developed patient-facing version of your child's test report. We are very interested in your opinions of that report for delivering genetic test information to patients and their families. This interview may take up to an hour, during which time I will ask questions about your experience with your child's genetic test results and how the results reports have been useful or not for you. Everything you tell me will be kept confidential. This means that your interview responses will only be shared with research team members. When we write our report on this and the other interviews we are conducting, nothing in our report will identify you. Please be honest with your responses. You can say whatever you want – nothing will hurt my feelings and nothing you say will have a negative effect on your care. Please remember: you don't have to talk about anything you don't want to talk about. You can decline to answer any question and you may end the interview at any time.

Discussion questions:

General questions:

1) Please describe for me your experience with receiving your child's test result and report

[Probe as necessary for information]

2) What did you find helpful about the computerized version of your child's genetic test report overall?
    - How many times did you access it?
    - What did you do with the information?
    - How have you used it so far? (did you print any part of it, show any part to your doctor, daycare provider, family members, etc)
    - IF NOT VIEWED/USED – why have you not used it/what is not helpful/useful about the information?

Report Overview Section:

3) What was helpful about this section for you?
4) What did you understand about your child's test result after reading that section?
5) What else did you need to see in that section? (what additional questions did you have after reading it)?

6) How often did you access that section?
7) What was not useful/too much in this section?
8) IF NOT VIEWED/USED – why have you not used it/what is not helpful/useful about the information?

Primary Findings Section:

1) What was helpful about this section for you?
2) What did you understand about your child's test result after reading that section?
3) What else did you need to see in that section? (what additional questions did you have after reading it)?
4) How often did you access that section?
5) What was not useful/too much in this section?
6) IF NOT VIEWED/USED – why have you not used it/what is not helpful/useful about the information?

Additional Findings Section (ASK ONLY IF patient had an additional finding)

1) What was helpful about this section for you?
2) What did you understand about your child's test result after reading that section?
3) What else did you need to see in that section? (what additional questions did you have after reading it)?
4) How often did you access that section?
5) What was not useful/too much in this section?
6) IF NOT VIEWED/USED – why have you not used it/what is not helpful/useful about the information?

Other sections (resources, glossary)

1) What was helpful about this section for you?
2) What did you understand about your child's test result after reading that section?
3) What else did you need to see in that section? (what additional questions did you have after reading it)?
4) How often did you access that section?
5) Did you follow any links in this section for more information? How did that work for you? How useful was it?
6) What was not useful/too much in this section?

7) IF NOT VIEWED/USED – why have you not used it/what is not helpful/useful about the information?

[FOR POSITIVE RESULTS ONLY]: PROGNOSTIC TABLE

1) What was helpful about this section for you?
2) What did you understand about your child's test result after reading that section?
3) What else did you need to see in that section? (what additional questions did you have after reading it)?
4) How often did you access that section?
5) What was not useful/too much in this section?
6) What did you do with the information in this section
    - Who have you shared this information with
    - What do you see yourself doing with this information in the future?
7) IF NOT VIEWED/USED – why have you not used it/what is not helpful/useful about the information?

Utility and Communication:

1) How have you used this report in your child's record when seeing your other healthcare providers?
    - Have you had them bring it up during a visit?
    - Have you directed them to it during a visit?
    - IF NOT USED – why have you not used it/what is not helpful/useful about the information?

2) How have you used this report with your child's school / family members?
    - How did you use it? (bring it up on a tablet/computer, printout, etc)
    - How has having this report changed how you explain your child's condition to other parents or family members?
    - How helpful was it? How did it help you achieve your goals by showing it to them?
    - IF NOT USED – why have you not shared with others?

3) How has having this report changed how you communicate with providers? With family? With others who care for your child?
    - What makes this information useful for you

4) What else do you need from this report?
    - What did not meet your expectations?

FINALLY, Is there anything I haven't asked you about that you think would be helpful for this research?

Thank you!!!
