# Supplementary material for: Impact of a Patient-Facing Enhanced Genomic Results Report to Improve Understanding, Engagement, and Communication
Source: J Genet Couns. 2017 Dec 4;27(2):358–69. doi: 10.1007/s10897-017-0176-6 (PMC5859697; doi:10.1007/s10897-017-0176-6)
Supplement: Supplementary file 4 — (PDF 795 kb) [file 10897_2017_176_MOESM4_ESM.pdf]

<u>Supplementary Table 1</u>: Survey results Intervention group at baseline and 3 months post report

	Baseline		3 Month	
	n	%	n	%
**Survey Respondents**	**21**		**21**	
Section 1: General Health				
1. In general how would you describe your health?				
Excellent	3	14.3%	5	23.8%
Very good	12	57.1%	11	52.4%
Good	3	14.3%	3	14.3%
Fair	3	14.3%	2	9.5%
Poor	0	0.0%	0	0.0%
2. Overall, how confident are you about your ability to take good care of your health?				
Completely confident	6	28.6%	9	42.9%
Very confident	12	57.1%	8	38.1%
Somewhat confident	3	14.3%	2	9.5%
A little confident	0	0.0%	2	9.5%
Not confident at all	0	0.0%	0	0.0%
5. In general, how would you describe your child's health?				
Excellent	3	14.3%	2	9.5%
Very good	3	14.3%	7	33.3%
Good	8	38.1%	3	14.3%
Fair	3	14.3%	7	33.3%
Poor	4	19.0%	2	9.5%
6. Overall, how confident are you about your ability to take good care of your child's health				
Completely confident	12	57.1%	9	42.9%
Very confident	5	23.8%	8	38.1%
Somewhat confident	3	14.3%	3	14.3%
A little confident	1	4.8%	1	4.8%
Not confident at all	0	0.0%	0	0.0%
Section 2: Health Information Preferences				
1. When you are dealing with health concerns for yourself of your child, how do you like to handle things?				
1. I like to gather as much information as I can before making a decision				
Not at all true	0	0.0%	0	0.0%
A little bit	2	9.5%	0	0.0%
Somewhat true	1	4.8%	2	9.5%

Quite a bit	6	28.6%	4	19.0%
Very much true	12	57.1%	15	71.4%
2. I like to review information multiple times before making a decision				
Not at all true	0	0.0%	0	0.0%
A little bit	2	9.5%	0	0.0%
Somewhat true	2	9.5%	5	23.8%
Quite a bit	8	38.1%	7	33.3%
Very much true	9	42.9%	9	42.9%
3. After I've made a decision, I continue to look for related information				
Not at all true	0	0.0%	0	0.0%
A little bit	3	14.3%	1	4.8%
Somewhat true	4	19.0%	4	19.0%
Quite a bit	6	28.6%	10	47.6%
Very much true	8	38.1%	6	28.6%
4. I like to make decisions quickly				
Not at all true	5	23.8%	8	38.1%
A little bit	7	33.3%	4	19.0%
Somewhat true	7	33.3%	7	33.3%
Quite a bit	1	4.8%	2	9.5%
Very much true	1	4.8%	0	0.0%
5. I have difficulty making sense of information from multiple sources				
Not at all true	12	57.1%	8	38.1%
A little bit	5	23.8%	4	19.0%
Somewhat true	2	9.5%	6	28.6%
Quite a bit	1	4.8%	2	9.5%
Very much true	1	4.8%	1	4.8%
Total	21		21	
6. I fear that I might find out something that I don't want to know				
Not at all true	6	30.0%	8	38.1%
A little bit	6	30.0%	6	28.6%
Somewhat true	3	15.0%	2	9.5%
Quite a bit	4	20.0%	1	4.8%
Very much true	1	5.0%	4	19.0%
Missing	*1*	*4.8%*	*0*	*0.0%*
7. I feel overwhelmed by the amount of information available				
Not at all true	12	60.0%	9	42.9%

A little bit	4	20.0%	7	33.3%
Somewhat true	3	15.0%	3	14.3%
Quite a bit	0	0.0%	1	4.8%
Very much true	1	5.0%	1	4.8%
Missing	*1*	*4.8%*	*0*	*0.0%*
8. I think it's the doctor's job to deal with the information, not mine				
Not at all true	15	71.4%	15	71.4%
A little bit	5	23.8%	5	23.8%
Somewhat true	1	4.8%	1	4.8%
Quite a bit	0	0.0%	0	0.0%
Very much true	0	0.0%	0	0.0%
HIOS Engagement Subscore, median (IQR)	3.00	(2.75, 3.75)	3.25	(2.75, 3.50)
HIOS Apprehension Subscore, median (IQR) *(One patient answered 2 of 4 subscale questions at baseline. Used average of their two responses for calculation.)*	0.75	(0.00, 1.00)	1.00	(0.50, 1.50)
2. Do you ask family members or friends for information or advice on health topics?				
Yes	14	66.7%	13	65.0%
No	7	33.3%	7	35.0%
Missing	*0*	*0.0%*	*1*	*4.8%*
3. Who do you ask for this information? *(of n = 16 who provided write-in responses at baseline)* *(of n = 12 who provided write-in responses at 3 months)*				
Spouse				
Yes	2	12.5%	3	25.0%
No	14	87.5%	9	75.0%
Family				
Yes	6	37.5%	3	25.0%
No	10	62.5%	9	75.0%
Friends				
Yes	2	12.5%	1	8.3%
No	14	87.5%	11	91.7%
Medical Professionals				
Yes	0	0.0%	0	0.0%
No	16	100.0%	12	100.0%
Support Groups				
Yes	1	6.3%	2	16.7%
No	15	93.8%	10	83.3%
Family/Friends with Medical Backgrounds				
Yes	8	50.0%	6	50.0%

No	8	50.0%	6	50.0%
Internet/Books				
Yes	1	6.3%	0	0.0%
No	15	93.8%	12	100.0%
Co-workers				
Yes	0	0.0%	0	0.0%
No	16	100.0%	12	100.0%
**Section 3: Internet use and information seeking**				
1. Have you ever looked for information about health or medical topics fos your child from any source?				
Yes	17	81.0%	17	81.0%
No	4	19.0%	4	19.0%
2 and 3. The most recent time you looked for information about health or medical topics for your child where did you go? *(of n = 17 at baseline and n = 17 at three months who responded "yes" to ever looking for health related information)*				
Books				
Yes	5	29.4%	10	58.8%
No	12	70.6%	7	41.2%
Brochures, pamphlets, ect.				
Yes	5	29.4%	8	47.1%
No	12	70.6%	9	52.9%
Cancer organization				
Yes	0	0.0%	2	11.8%
No	17	100.0%	15	88.2%
Family				
Yes	4	23.5%	7	41.2%
No	13	76.5%	10	58.8%
Friend/Co-worker				
Yes	4	23.5%	4	23.5%
No	13	76.5%	13	76.5%
Doctor or health care provider				
Yes	6	35.3%	11	64.7%
No	11	64.7%	6	35.3%
Internet				
Yes	14	82.4%	15	88.2%
No	3	17.6%	2	11.8%
Library				
Yes	0	0.0%	3	17.6%
No	17	100.0%	14	82.4%

Magazines				
Yes	4	23.5%	5	29.4%
No	13	76.5%	12	70.6%
Newspapers				
Yes	3	17.6%	4	23.5%
No	14	82.4%	13	76.5%
Telephone information number				
Yes	0	0.0%	2	11.8%
No	17	100.0%	15	88.2%
Complementary, alternative, or unconventional practitioner				
Yes	1	5.9%	4	23.5%
No	16	94.1%	13	76.5%
Genetic test report				
Yes	3	17.6%	6	35.3%
No	14	82.4%	11	64.7%
4. Based on the results of your most recent search for information about health or medical topics regarding your child, how much do you agree or disagree with each of the following? *(of n = 17 at baseline and n = 17 at three months who responded "yes" to ever looking for health related information)*				
It took a lot of effort to get the information you needed				
Strongly agree	2	11.8%	4	25.0%
Somewhat agree	4	23.5%	5	31.3%
Somewhat disagree	9	52.9%	7	43.8%
Strongly disagree	2	11.8%	0	0.0%
Missing	*0*	*0.0%*	*1*	*5.9%*
You felt frustrated during your search for the information				
Strongly agree	1	6.7%	2	12.5%
Somewhat agree	6	40.0%	8	50.0%
Somewhat disagree	6	40.0%	5	31.3%
Strongly disagree	2	13.3%	1	6.3%
Missing	*2*	*11.8%*	*1*	*5.9%*
You were concerned about the quality of the information				
Strongly agree	5	33.3%	2	12.5%
Somewhat agree	5	33.3%	9	56.3%
Somewhat disagree	4	26.7%	3	18.8%
Strongly disagree	1	6.7%	2	12.5%
Missing	*2*	*11.8%*	*1*	*5.9%*
The information you found was hard to understand				

Strongly agree	0	0.0%	0	0.0%
Somewhat agree	4	26.7%	5	29.4%
Somewhat disagree	9	60.0%	7	41.2%
Strongly disagree	2	13.3%	5	29.4%
Missing	*2*	*11.8%*	*0*	*0.0%*
5. Overall, how confident are you that you can get advice or information about health and medical topics if you needed it?				
Completely confident	7	33.3%	5	25.0%
Very confident	8	38.1%	11	55.0%
Somewhat confident	6	28.6%	4	20.0%
A little confident	0	0.0%	0	0.0%
Not confident at all	0	0.0%	0	0.0%
Missing	*0*	*0.0%*	*1*	*4.8%*
**Section 4: Provider Communication**				
1. In general how often do you do each of the following?				
Take with you to your child's doctor visits a list of questions or concerns you want to cover				
Always	9	45.0%	9	47.4%
Usually	2	10.0%	5	26.3%
Sometimes	8	40.0%	4	21.1%
Never	1	5.0%	1	5.3%
Missing	*1*	*4.8%*	*2*	*9.5%*
Take a list of all your child's prescribed medicines to his/her doctor visits				
Always	6	31.6%	7	38.9%
Usually	3	15.8%	4	22.2%
Sometimes	2	10.5%	3	16.7%
Never	8	42.1%	4	22.2%
Missing	*2*	*9.5%*	*3*	*14.3%*
Ask your child's doctor to explain a test, treatment, or procedure to you in detail				
Always	11	55.0%	14	73.7%
Usually	4	20.0%	4	21.1%
Sometimes	4	20.0%	1	5.3%
Never	1	5.0%	0	0.0%
Missing	*1*	*4.8%*	*2*	*9.5%*
Read information about a new prescription, such as side effects and precautions				
Always	15	75.0%	14	73.7%
Usually	1	5.0%	4	21.1%
Sometimes	2	10.0%	1	5.3%

Never	2	10.0%	0	0.0%
Missing	*1*	*4.8%*	*2*	*9.5%*
Do you own research on a health or medical topic after seeing your child's doctor				
Always	11	55.0%	8	42.1%
Usually	2	10.0%	7	36.8%
Sometimes	6	30.0%	4	21.1%
Never	1	5.0%	0	0.0%
Missing	*1*	*4.8%*	*2*	*9.5%*
Take with you to your child's doctor visit any kind of health information you have found				
Always	3	15.0%	6	31.6%
Usually	1	5.0%	5	26.3%
Sometimes	13	65.0%	5	26.3%
Never	3	15.0%	3	15.8%
Missing	*1*	*4.8%*	*2*	*9.5%*
2. Have you ever talked to your child's doctor, nurse, or other health care provider about any kind of health information you have gotten from the internet?				
Yes	15	71.4%	14	66.7%
No	6	28.6%	7	33.3%
3. When you talked with your child's health care provider, how interested were they in hearing about the information you found online? *(of n = 15 parents at baseline and n = 14 at three months who responded "yes" to ever talking to provider about info found on internet)*				
Very interested	4	28.6%	5	38.5%
Somewhat interested	5	35.7%	4	30.8%
A little interested	4	28.6%	4	30.8%
Not at all interested	1	7.1%	0	0.0%
Missing	*1*	*6.7%*	*1*	*7.1%*
4. The following questions are about your communication with your child's doctors, nurses, or other health professionals you saw during the last 12 months. How often did they do each of the following? *(of n = 15 parents at baseline and n = 14 at three months who responded "yes" to ever talking to provider about info found on internet)*				
Give you the chance to ask all the health-related questions you had				
Always	11	78.6%	9	69.2%

Usually	3	21.4%	2	15.4%
Sometimes	0	0.0%	2	15.4%
Never	0	0.0%	0	0.0%
Missing	*1*	*6.7%*	*1*	*7.1%*
Give the attention you needed to your feelings and emotions				
Always	10	71.4%	7	53.8%
Usually	2	14.3%	3	23.1%
Sometimes	2	14.3%	2	15.4%
Never	0	0.0%	1	7.7%
Missing	*1*	*6.7%*	*1*	*7.1%*
Involve you in the decisions about your child's health care as much as you wanted				
Always	12	85.7%	10	76.9%
Usually	1	7.1%	0	0.0%
Sometimes	1	7.1%	2	15.4%
Never	0	0.0%	1	7.7%
Missing	*1*	*6.7%*	*1*	*7.1%*
Make sure you understood the things you needed to do to take care of your child's health				
Always	11	78.6%	10	76.9%
Usually	1	7.1%	0	0.0%
Sometimes	2	14.3%	2	15.4%
Never	0	0.0%	1	7.7%
Missing	*1*	*6.7%*	*1*	*7.1%*
Explain things in a way that you could understand				
Always	10	71.4%	9	69.2%
Usually	3	21.4%	1	7.7%
Sometimes	1	7.1%	3	23.1%
Never	0	0.0%	0	0.0%
Missing	*1*	*6.7%*	*1*	*7.1%*
Spend enough time with you				
Always	9	64.3%	9	69.2%
Usually	2	14.3%	1	7.7%
Sometimes	3	21.4%	2	15.4%
Never	0	0.0%	1	7.7%
Missing	*1*	*6.7%*	*1*	*7.1%*
Help you deal with feelings of uncertainty about your child's health or health care				
Always	9	64.3%	8	61.5%
Usually	2	14.3%	2	15.4%

Sometimes	2	14.3%	2	15.4%
Never	1	7.1%	1	7.7%
Missing	*1*	*6.7%*	*1*	*7.1%*
5. Overall, how would you rate the quality of health care your child received in the past 12 months (baseline survey) or past 3 months (three month survey)				
Excellent	12	57.1%	12	57.1%
Very good	3	14.3%	5	23.8%
Good	5	23.8%	3	14.3%
Fair	1	4.8%	0	0.0%
Poor	0	0.0%	1	4.8%
In the past 12 months (baseline survey) or 3 months (three month survey), how often did you feel you could rely on your doctors, nurses, or other health care professionals to take care of your child's health care needs?				
Always	10	47.6%	11	52.4%
Usually	7	33.3%	7	33.3%
Sometimes	4	19.0%	2	9.5%
Never	0	0.0%	1	4.8%
**Section 5: Participating in the Whole Genome Sequencing Research Study**				
Sometime people feel differently about genetic testing decisions after they receive results. When you think about having gone through the whole genome testing for your child and the results you received, how much would you agree or disagree with the following statements?				
1. It was the right decision				
Strongly agree	15	71.4%	13	61.9%
Agree	3	14.3%	5	23.8%
Neither agree nor disagree	2	9.5%	1	4.8%
Disagree	1	4.8%	0	0.0%
Strongly disagree	0	0.0%	2	9.5%
Total	21		21	
2. I regret the choice that was made				
Strongly agree	1	4.8%	1	4.8%
Agree	0	0.0%	0	0.0%
Neither agree nor disagree	2	9.5%	3	14.3%
Disagree	6	28.6%	2	9.5%
Strongly disagree	12	57.1%	15	71.4%
3. I would go for the same choice if I had to do it over again				

Strongly agree	15	71.4%	16	76.2%
Agree	3	14.3%	5	23.8%
Neither agree nor disagree	1	4.8%	0	0.0%
Disagree	2	9.5%	0	0.0%
Strongly disagree	0	0.0%	0	0.0%
4. The choice did me a lot of harm				
Strongly agree	1	4.8%	0	0.0%
Agree	0	0.0%	0	0.0%
Neither agree nor disagree	0	0.0%	2	9.5%
Disagree	4	19.0%	2	9.5%
Strongly disagree	16	76.2%	17	81.0%
5. The decision was a wise one				
Strongly agree	13	61.9%	14	66.7%
Agree	4	19.0%	3	14.3%
Neither agree nor disagree	4	19.0%	3	14.3%
Disagree	0	0.0%	0	0.0%
Strongly disagree	0	0.0%	1	4.8%
Decision Regret Score, median (IQR)	5	0, 25	0	(0, 20)
Decision Regret Score				
0	10	47.6%	12	57.1%
> 0	11	52.4%	9	42.9%
Decision Regret Score				
Expressed regret on at least one of 5 questions	5	23.8%	7	33.3%
No regret expressed on any questions	16	76.2%	14	66.7%
Decision Regret Score for Those with Scores > 0, median (IQR)<br>*n = 11 baseline scores ranging from 5 to 80*<br>*n = 9 three month scores ranging from 15 to 60*	25	(5.0, 40.0)	25	(15, 35)
**Section 6: Response to whole genome sequencing results (None of the respondents had positive results)**				
1. I understood how my child came to have this gene	N/A	N/A	N/A	N/A
2. I understand the health risks my relatives face because of my child having the gene	N/A	N/A	N/A	N/A
3. I feel certain that I undertand the meaning of my child having this gene	N/A	N/A	N/A	N/A
4. I understand the chances I have of passing this gene along to other children	N/A	N/A	N/A	N/A
5. I feel that I can explain to other people what my child having this gene means	N/A	N/A	N/A	N/A
PAGIS Certainty Score, median (IQR)	N/A	N/A	N/A	N/A
**Section 7: Response to the whole genome sequencing results**				

The statements below reflect responses some people have after learning a child's genetic test result. Please respond how much each of these statements represents how you have felt in the past week. In the past week how often have you been?				
1. Feeling upset about your child's test results?				
Never	12	70.6%	11	57.9%
Rarely	3	17.6%	4	21.1%
Sometimes	0	0.0%	2	10.5%
Often	2	11.8%	2	10.5%
Missing	*4*	*19.0%*	*2*	*9.5%*
2. Feeling sad about your child's test results?				
Never	10	62.5%	12	63.2%
Rarely	3	18.8%	3	15.8%
Sometimes	1	6.3%	2	10.5%
Often	2	12.5%	2	10.5%
Missing	*5*	*23.8%*	*2*	*9.5%*
3. Feeling anxious or nervous about your child's test results?				
Never	11	64.7%	13	68.4%
Rarely	4	23.5%	3	15.8%
Sometimes	1	5.9%	1	5.3%
Often	1	5.9%	2	10.5%
Missing	*4*	*19.0%*	*2*	*9.5%*
4. Feeling guilty about your child's test results?				
Never	12	70.6%	13	68.4%
Rarely	2	11.8%	4	21.1%
Sometimes	3	17.6%	1	5.3%
Often	0	0.0%	1	5.3%
Missing	*4*	*19.0%*	*2*	*9.5%*
5. Feeling relieved about your child's test results?				
Never	6	40.0%	6	37.5%
Rarely	5	33.3%	3	18.8%
Sometimes	2	13.3%	4	25.0%
Often	2	13.3%	3	18.8%
Missing	*6*	*28.6%*	*5*	*23.8%*
6. Feeling happy about your child's test results?				
Never	5	35.7%	5	33.3%
Rarely	5	35.7%	5	33.3%
Sometimes	3	21.4%	2	13.3%
Often	1	7.1%	3	20.0%

Missing	*7*	*33.3%*	*6*	*28.6%*
7. Feeling loss of control				
Never	9	52.9%	12	63.2%
Rarely	4	23.5%	1	5.3%
Sometimes	3	17.6%	4	21.1%
Often	1	5.9%	2	10.5%
Missing	*4*	*19.0%*	*2*	*9.5%*
8. Having problems enjoying your life because of your child's results				
Never	11	61.1%	14	77.8%
Rarely	5	27.8%	2	11.1%
Sometimes	2	11.1%	1	5.6%
Often	0	0.0%	1	5.6%
Missing	*3*	*14.3%*	*3*	*14.3%*
9. Worring about your child's risk of becoming sick or ill				
Never	6	35.3%	7	36.8%
Rarely	2	11.8%	3	15.8%
Sometimes	4	23.5%	3	15.8%
Often	5	29.4%	6	31.6%
Missing	*4*	*19.0%*	*2*	*9.5%*
10. Being uncertain about what your child's results mean about your child's future health				
Never	6	35.3%	7	41.2%
Rarely	1	5.9%	2	11.8%
Sometimes	4	23.5%	3	17.6%
Often	6	35.3%	5	29.4%
Missing	*4*	*19.0%*	*4*	*19.0%*
11. Being uncertain about what your child's results mean for any other children and/or your family's health future				
Never	7	41.2%	10	55.6%
Rarely	2	11.8%	2	11.1%
Sometimes	4	23.5%	3	16.7%
Often	4	23.5%	3	16.7%
Missing	*4*	*19.0%*	*3*	*14.3%*
12. Having difficulty making decisions about health screening or disease prevention for your child				
Never	13	76.5%	12	66.7%
Rarely	1	5.9%	0	0.0%
Sometimes	2	11.8%	3	16.7%
Often	1	5.9%	3	16.7%
Missing	*4*	*19.0%*	*3*	*14.3%*

13. Understanding clearly your child's choices for health screening or disease prevention				
Never	8	47.1%	6	33.3%
Rarely	2	11.8%	1	5.6%
Sometimes	3	17.6%	4	22.2%
Often	4	23.5%	7	38.9%
Missing	*4*	*19.0%*	*3*	*14.3%*
14. Feeling frustrated that there are no definite health guidelines for your child				
Never	5	29.4%	5	26.3%
Rarely	1	5.9%	0	0.0%
Sometimes	1	5.9%	5	26.3%
Often	10	58.8%	9	47.4%
Missing	*4*	*19.0%*	*2*	*9.5%*
15. Thinking about your child's result has affected your work or family life				
Never	9	52.9%	15	78.9%
Rarely	2	11.8%	0	0.0%
Sometimes	4	23.5%	3	15.8%
Often	2	11.8%	1	5.3%
Missing	*4*	*19.0%*	*2*	*9.5%*
16. Feeling concerned about how your child's results will affect your health insurance status				
Never	14	82.4%	12	63.2%
Rarely	3	17.6%	4	21.1%
Sometimes	0	0.0%	2	10.5%
Often	0	0.0%	1	5.3%
Missing	*4*	*19.0%*	*2*	*9.5%*
17. Having difficulty talking about your child's results with family members				
Never	12	70.6%	16	84.2%
Rarely	0	0.0%	2	10.5%
Sometimes	4	23.5%	1	5.3%
Often	1	5.9%	0	0.0%
Missing	*4*	*19.0%*	*2*	*9.5%*
18. Feeling that your family has been supportive during the process of learning the genetic result				
Never	5	29.4%	5	29.4%
Rarely	2	11.8%	4	23.5%
Sometimes	3	17.6%	1	5.9%
Often	7	41.2%	7	41.2%
Missing	*4*	*19.0%*	*4*	*19.0%*

19. Feeling satisfied with family communication about your child's genetic results				
Never	4	23.5%	5	31.3%
Rarely	3	17.6%	3	18.8%
Sometimes	3	17.6%	1	6.3%
Often	7	41.2%	7	43.8%
Missing	*4*	*19.0%*	*5*	*23.8%*
20. Worrying that your child's results have brought about conflict within your family				
Never	14	77.8%	14	82.4%
Rarely	4	22.2%	2	11.8%
Sometimes	0	0.0%	0	0.0%
Often	0	0.0%	1	5.9%
Missing	*3*	*14.3%*	*4*	*19.0%*
21. Feeling regret about getting the test result				
Never	17	94.4%	13	81.3%
Rarely	1	5.6%	2	12.5%
Sometimes	0	0.0%	0	0.0%
Often	0	0.0%	1	6.3%
Missing	*3*	*14.3%*	*5*	*23.8%*
22. Feeling that the genetic test result has made it harder to cope with my child's diagnosis				
Never	15	83.3%	12	70.6%
Rarely	2	11.1%	4	23.5%
Sometimes	1	5.6%	0	0.0%
Often	0	0.0%	1	5.9%
Missing	*3*	*14.3%*	*4*	*19.0%*
23. Feeling that the genetic test result has made it easier to cope with my child's diagnosis				
Never	12	75.0%	7	46.7%
Rarely	1	6.3%	5	33.3%
Sometimes	1	6.3%	3	20.0%
Often	2	12.5%	0	0.0%
Missing	*5*	*23.8%*	*6*	*28.6%*
MICRA Distress Subscale Raw Score, (all Qs answered) median (IQR) *(n missing baseline = 5, n missing 3 month = 3)*	2.5	(0.0, 8.0)	0.0	(0.0, 10.0)
MICRA Distress Subscale Scaled Score (all Qs answered), median (IQR) *(n missing baseline = 5, n missing 3 month = 3)*	8.3	(0.0, 26.7)	0.0	(0.0, 33.3)

MICRA Distress Subscale Scaled Score (at least 1 Q answered), median (IQR)<br>*(n missing baseline = 3, n missing 3 month = 2)*	5.8	(0.0, 20.0)	0.0	(0.0, 33.3)
MICRA Uncertainty Subscale Raw Score (all Qs answered), median (IQR)<br>*(n missing baseline= 4, n missing 3 month = 5)*	13.0	(5.0, 21.0)	9.0	(3.5, 25.0)
MICRA Uncertainty Subscale Scaled Score (all Qs answered), median (IQR)<br>*(n missing baseline= 4, n missing 3 month = 5)*	28.9	(11.1, 46.7)	20.0	(7.8, 55.6)
MICRA Uncertainty Subscale Scaled Score (at least 1 Q answered), median (IQR)<br>*(n missing baseline= 3, n missing 3 month = 2)*	28.9	(11.1, 46.7)	20.0	(13.3, 55.6)
MICRA Positive Experience Subscale Raw Score (all Qs answered), median (IQR)<br>*(n missing baseline = 7, n missing 3 month =6)*	12.0	(6.0, 14.0)	10.0	(2.0, 20.0)
MICRA Positive Experience Subscale Scaled Score (all Qs answered), median (IQR)<br>*(n missing baseline = 7, n missing 3 month =6)*	60.0	(30.0, 70.0)	50.0	(10.0, 100.0)
MICRA Positive Experience Subscale Scaled Score (at least 1 Q answered), median (IQR)<br>*(n missing baseline = 4, n missing 3 month =4)*	50.0	(10.0, 70.0)	50.0	(0.0, 100.0)
MICRA Overall (Qs 1-21) Raw Score (all Qs answered), median (IQR)<br>*(n missing baseline = 7, n missing 3 month = 7)*	31.0	(25.0, 40.0)	27.5	(7.0, 50.0)
MICRA Overall (Qs 1-21) Scaled Score (all Qs answered), median (IQR)<br>*(n missing baseline = 7, n missing 3 month = 7)*	29.5	(23.8, 38.1)	26.2	(6.7, 47.6)
MICRA Overall (Qs 1-21) Scaled Score (at least 1 Q answered), median (IQR)<br>*(n missing baseline = 3, n missing 3 month = 3)*	29.0	(14.3, 35.4)	22.0	(6.7, 46.7)
**Section 8: Questions about the report you received on your child's genetic testing**				
1. Did you read the letter describing your child's whole genome sequencing results?				
Yes	12	66.7%	17	85.0%
No	6	33.3%	3	15.0%
Missing	*3*	*14.3%*	*1*	*4.8%*
2. How helpful were the various parts of the genetic testing report you received? *(of n = 12 at baseline and n = 17 at three months who said they read the letter)*				
The summary				
Not helpful	1	10.0%	4	33.3%

A little bit helpfu	3	30.0%	2	16.7%
Somewhat helpful	3	30.0%	2	16.7%
Quite a bit helpful	3	30.0%	3	25.0%
Very helpful	0	0.0%	1	8.3%
Missing	*2*	*16.7%*	*5*	*29.4%*
The detailed explanation				
Not helpful	2	20.0%	4	33.3%
A little bit helpfu	2	20.0%	2	16.7%
Somewhat helpful	3	30.0%	2	16.7%
Quite a bit helpful	1	10.0%	1	8.3%
Very helpful	2	20.0%	3	25.0%
Missing	*2*	*16.7%*	*5*	*29.4%*
The care instructions				
Not helpful	1	11.1%	6	54.5%
A little bit helpfu	2	22.2%	1	9.1%
Somewhat helpful	5	55.6%	3	27.3%
Quite a bit helpful	1	11.1%	0	0.0%
Very helpful	0	0.0%	1	9.1%
Missing	*3*	*25.0%*	*6*	*35.3%*
3. Did you review the genetic testing report you received with your provider? *(of n = 12 at baseline and n = 17 at three months who said they read the letter)*				
Yes, in person	9	81.8%	11	64.7%
Yes, over the phone	0	0.0%	0	0.0%
No	2	18.2%	6	35.3%
4. Did reviewing the genetic testing report with your provider help improve your understanding of your child's health condition? *(of n = 9 at baseline and n = 11 at three months who said they read the letter with their provider)*				
n/a No, I already understood everything I needed	3	33.3%	2	20.0%
Didn't help	2	22.2%	6	60.0%
Helped a little	1	11.1%	1	10.0%
Helped some	1	11.1%	0	0.0%
Helped a fair amount	0	0.0%	1	10.0%
Helped a great deal	2	22.2%	0	0.0%
Missing	*0*	*0.0%*	*1*	*9.1%*

<u>Supplementary Table 2:</u> Survey results Usual Care – results from Baseline, 3 months post baseline and 6 months post baseline (3 months post-report)

	Baseline		3 Month		6 Month	
	n	%	n	%	n	%
**Survey Respondents**	**15**		**15**		**15**	
Section 1: General Health						
1. In general how would you describe your health?						
Excellent	3	20.0%	3	20.0%	3	20.0%
Very good	6	40.0%	4	26.7%	4	26.7%
Good	5	33.3%	6	40.0%	7	46.7%
Fair	1	6.7%	1	6.7%	1	6.7%
Poor	0	0.0%	1	6.7%	0	0.0%
2. Overall, how confident are you about your ability to take good care of your health?						
Completely confident	4	26.7%	5	33.3%	5	33.3%
Very confident	8	53.3%	7	46.7%	8	53.3%
Somewhat confident	2	13.3%	3	20.0%	2	13.3%
A little confident	1	6.7%	0	0.0%	0	0.0%
Not confident at all	0	0.0%	0	0.0%	0	0.0%
5. In general, how would you describe your child's health?						
Excellent	2	13.3%	2	13.3%	1	6.7%
Very good	6	40.0%	6	40.0%	5	33.3%
Good	5	33.3%	5	33.3%	5	33.3%
Fair	1	6.7%	1	6.7%	3	20.0%
Poor	1	6.7%	1	6.7%	1	6.7%
6. Overall, how confident are you about your ability to take good care of your child's health						
Completely confident	4	26.7%	4	26.7%	5	33.3%
Very confident	6	40.0%	6	40.0%	8	53.3%
Somewhat confident	3	20.0%	3	20.0%	2	13.3%
A little confident	1	6.7%	2	13.3%	0	0.0%
Not confident at all	1	6.7%	0	0.0%	0	0.0%
Section 2: Health Information Preferences						
1. When you are dealing with health concerns for yourself of your child, how do you like to handle things?						

1. I like to gather as much information as I can before making a decision						
Not at all true	0	0.0%	0	0.0%	0	0.0%
A little bit	1	6.7%	0	0.0%	1	6.7%
Somewhat true	0	0.0%	1	6.7%	2	13.3%
Quite a bit	5	33.3%	3	20.0%	4	26.7%
Very much true	9	60.0%	11	73.3%	8	53.3%
2. I like to review information multiple times before making a decision						
Not at all true	0	0.0%	0	0.0%	0	0.0%
A little bit	1	6.7%	0	0.0%	2	13.3%
Somewhat true	3	20.0%	4	26.7%	3	20.0%
Quite a bit	4	26.7%	5	33.3%	6	40.0%
Very much true	7	46.7%	6	40.0%	4	26.7%
3. After I've made a decision, I continue to look for related information						
Not at all true	1	6.7%	1	6.7%	0	0.0%
A little bit	3	20.0%	0	0.0%	2	13.3%
Somewhat true	4	26.7%	4	26.7%	5	33.3%
Quite a bit	4	26.7%	7	46.7%	4	26.7%
Very much true	3	20.0%	3	20.0%	4	26.7%
4. I like to make decisions quickly						
Not at all true	5	33.3%	7	50.0%	7	50.0%
A little bit	7	46.7%	1	7.1%	1	7.1%
Somewhat true	3	20.0%	3	21.4%	5	35.7%
Quite a bit	0	0.0%	3	21.4%	1	7.1%
Very much true	0	0.0%	0	0.0%	0	0.0%
Missing	*0*	*0.0%*	*1*	*6.7%*	*1*	*6.7%*
5. I have difficulty making sense of information from multiple sources						
Not at all true	4	26.7%	4	26.7%	4	26.7%
A little bit	4	26.7%	6	40.0%	5	33.3%
Somewhat true	6	40.0%	4	26.7%	5	33.3%
Quite a bit	1	6.7%	1	6.7%	1	6.7%
Very much true	0	0.0%	0	0.0%	0	0.0%
6. I fear that I might find out something that I don't want to know						
Not at all true	7	46.7%	7	46.7%	8	53.3%
A little bit	3	20.0%	3	20.0%	4	26.7%

Somewhat true	5	33.3%	4	26.7%	1	6.7%
Quite a bit	0	0.0%	0	0.0%	2	13.3%
Very much true	0	0.0%	1	6.7%	0	0.0%
Missing	*0*	*0.0%*	*0*	*0.0%*	*0*	*0.0%*
7. I feel overwhelmed by the amount of information available						
Not at all true	5	33.3%	5	33.3%	7	46.7%
A little bit	3	20.0%	4	26.7%	1	6.7%
Somewhat true	4	26.7%	2	13.3%	3	20.0%
Quite a bit	2	13.3%	1	6.7%	4	26.7%
Very much true	1	6.7%	3	20.0%	0	0.0%
Missing	*0*	*0.0%*	*0*	*0.0%*	*0*	*0.0%*
8. I think it's the doctor's job to deal with the information, not mine						
Not at all true	8	53.3%	12	80.0%	10	66.7%
A little bit	3	20.0%	1	6.7%	1	6.7%
Somewhat true	3	20.0%	0	0.0%	3	20.0%
Quite a bit	1	6.7%	2	13.3%	1	6.7%
Very much true	0	0.0%	0	0.0%	0	0.0%
HIOS Engagement Subscore, median (IQR) *(One patient answered 3 of 4 app subscale questions for the 3 month survey and another answered 3 of 4 on the six month survey. Used average of their three responses for calculations.)*	3.00	(2.50, 3.50)	3.25	(2.25, 3.50)	3.25	(2.25, 4.00)
HIOS Apprehension Subscore, median (IQR)	0.75	(0.50, 2.00)	0.75	(0.25, 1.50)	1.00	(0.25, 1.50)
2. Do you ask family members or friends for information or advice on health topics?						
Yes	10	66.7%	12	80.0%	10	66.7%
No	5	33.3%	3	20.0%	5	33.3%
Missing	*0*	*0.0%*	*1*	*6.7%*	*1*	*6.7%*
3. Who do you ask for this information? *(of n = 11 who provided write-in responses at baseline)* *(of n = 13 who provided write-in responses at 3 months)* *(of n = 11 who provided write-in responses at 6 months)*						

Spouse						
Yes	2	18.2%	4	30.8%	4	36.4%
No	9	81.8%	9	69.2%	7	63.6%
Family						
Yes	3	27.3%	4	30.8%	3	27.3%
No	8	72.7%	9	69.2%	8	72.7%
Friends						
Yes	1	9.1%	4	30.8%	5	45.5%
No	10	90.9%	9	69.2%	6	54.5%
Medical Professionals						
Yes	5	45.5%	4	30.8%	5	45.5%
No	6	54.5%	9	69.2%	6	54.5%
Support Groups						
Yes	2	18.2%	2	15.4%	2	18.2%
No	9	81.8%	11	84.6%	9	81.8%
Family/Friends with Medical Backgrounds						
Yes	2	18.2%	2	15.4%	1	9.1%
No	9	81.8%	11	84.6%	10	90.9%
Internet/Books						
Yes	1	9.1%	3	23.1%	0	0.0%
No	10	90.9%	10	76.9%	11	100.0%
Co-workers						
Yes	2	18.2%	1	7.7%	0	0.0%
No	9	81.8%	12	92.3%	11	100.0%
**Section 3: Internet use and information seeking**						
1. Have you ever looked for information about health or medical topics fos your child from any source?						
Yes	13	86.7%	14	93.3%	13	86.7%
No	2	13.3%	1	6.7%	2	13.3%
Missing	*0*	*0.0%*	*0*	*0.0%*	*0*	*0.0%*
2 and 3. The most recent time you looked for information about health or medical topics for your child where did you go? *(of n = 13 at baseline, n = 14 at three months and n = 13 at six months who responded "yes" to ever looking for health related information)*						
Books						

Yes	1	7.7%	5	35.7%	2	15.4%
No	12	92.3%	9	64.3%	11	84.6%
Brochures, pamphlets, ect.						
Yes	1	7.7%	3	21.4%	0	0.0%
No	12	92.3%	11	78.6%	13	100.0%
Cancer organization						
Yes	1	7.7%	2	14.3%	0	0.0%
No	12	92.3%	12	85.7%	13	100.0%
Family						
Yes	3	23.1%	3	21.4%	0	0.0%
No	10	76.9%	11	78.6%	13	100.0%
Friend/Co-worker						
Yes	3	23.1%	3	21.4%	2	15.4%
No	10	76.9%	11	78.6%	11	84.6%
Doctor or health care provider						
Yes	6	46.2%	7	50.0%	6	46.2%
No	7	53.8%	7	50.0%	7	53.8%
Internet						
Yes	12	92.3%	13	92.9%	13	100.0%
No	1	7.7%	1	7.1%	0	0.0%
Library						
Yes	0	0.0%	0	0.0%	1	7.7%
No	13	100.0%	14	100.0%	12	92.3%
Magazines						
Yes	0	0.0%	2	14.3%	1	7.7%
No	13	100.0%	12	85.7%	12	92.3%
Newspapers						
Yes	0	0.0%	2	14.3%	0	0.0%
No	13	100.0%	12	85.7%	13	100.0%
Telephone information number						
Yes	0	0.0%	1	7.1%	0	0.0%
No	13	100.0%	13	92.9%	13	100.0%
Complementary, alternative, or unconventional practitioner						
Yes	0	0.0%	1	7.1%	0	0.0%
No	13	100.0%	13	92.9%	13	100.0%
Genetic test report						
Yes	0	0.0%	2	14.3%	0	0.0%
No	13	100.0%	12	85.7%	13	100.0%

4. Based on the results of your most recent search for information about health or medical topics regarding your child, how much do you agree or disagree with each of the following? *(of n = 13 at baseline, n = 14 at three months and n = 13 at six months who responded "yes" to ever looking for health related information)*						
It took a lot of effort to get the information you needed						
Strongly agree	1	7.7%	3	21.4%	2	15.4%
Somewhat agree	5	38.5%	4	28.6%	4	30.8%
Somewhat disagree	5	38.5%	7	50.0%	6	46.2%
Strongly disagree	2	15.4%	0	0.0%	1	7.7%
Missing	*0*	*0.0%*	*0*	*0.0%*	*0*	*0.0%*
You felt frustrated during your search for the information						
Strongly agree	3	23.1%	3	21.4%	2	15.4%
Somewhat agree	5	38.5%	3	21.4%	5	38.5%
Somewhat disagree	2	15.4%	5	35.7%	6	46.2%
Strongly disagree	3	23.1%	3	21.4%	0	0.0%
Missing	*2*	*13.3%*	*0*	*0.0%*	*0*	*0.0%*
You were concerned about the quality of the information						
Strongly agree	1	7.7%	3	21.4%	3	23.1%
Somewhat agree	9	69.2%	6	42.9%	4	30.8%
Somewhat disagree	0	0.0%	3	21.4%	4	30.8%
Strongly disagree	3	23.1%	2	14.3%	2	15.4%
Missing	*2*	*13.3%*	*0*	*0.0%*	*0*	*0.0%*
The information you found was hard to understand						
Strongly agree	0	0.0%	2	14.3%	0	0.0%
Somewhat agree	4	30.8%	5	35.7%	5	38.5%
Somewhat disagree	3	23.1%	3	21.4%	4	30.8%
Strongly disagree	6	46.2%	4	28.6%	4	30.8%
Missing	*2*	*13.3%*	*0*	*0.0%*	*0*	*0.0%*
5. Overall, how confident are you that you can get advice or information about health and medical topics if you needed it?						
Completely confident	4	28.6%	3	20.0%	5	33.3%
Very confident	3	21.4%	5	33.3%	4	26.7%

Somewhat confident	7	50.0%	7	46.7%	6	40.0%
A little confident	0	0.0%	0	0.0%	0	0.0%
Not confident at all	0	0.0%	0	0.0%	0	0.0%
Missing	*1*	*6.7%*	*0*	*0.0%*	*0*	*0.0%*
**Section 4: Provider Communication**						
1. In general how often do you do each of the following?						
Take with you to your child's doctor visits a list of questions or concerns you want to cover						
Always	3	20.0%	5	33.3%	1	6.7%
Usually	2	13.3%	4	26.7%	6	40.0%
Sometimes	6	40.0%	2	13.3%	5	33.3%
Never	4	26.7%	4	26.7%	3	20.0%
Missing	*0*	*0.0%*	*0*	*0.0%*	*0*	*0.0%*
Take a list of all your child's prescribed medicines to his/her doctor visits						
Always	5	33.3%	6	40.0%	3	20.0%
Usually	2	13.3%	3	20.0%	2	13.3%
Sometimes	2	13.3%	2	13.3%	6	40.0%
Never	6	40.0%	4	26.7%	4	26.7%
Missing	*0*	*0.0%*	*0*	*0.0%*	*0*	*0.0%*
Ask your child's doctor to explain a test, treatment, or procedure to you in detail						
Always	7	46.7%	4	26.7%	4	26.7%
Usually	1	6.7%	6	40.0%	5	33.3%
Sometimes	6	40.0%	5	33.3%	5	33.3%
Never	1	6.7%	0	0.0%	1	6.7%
Missing	*0*	*0.0%*	*0*	*0.0%*	*0*	*0.0%*
Read information about a new prescription, such as side effects and precautions						
Always	7	46.7%	4	26.7%	8	53.3%
Usually	2	13.3%	7	46.7%	3	20.0%
Sometimes	5	33.3%	3	20.0%	3	20.0%
Never	1	6.7%	1	6.7%	1	6.7%
Missing	*0*	*0.0%*	*0*	*0.0%*	*0*	*0.0%*
Do you own research on a health or medical topic after seeing your child's doctor						
Always	5	33.3%	4	26.7%	5	33.3%

Usually	2	13.3%	3	20.0%	3	20.0%
Sometimes	6	40.0%	7	46.7%	7	46.7%
Never	2	13.3%	1	6.7%	0	0.0%
Missing	*0*	*0.0%*	*0*	*0.0%*	*0*	*0.0%*
Take with you to your child's doctor visit any kind of health information you have found						
Always	3	20.0%	2	13.3%	1	6.7%
Usually	3	20.0%	4	26.7%	5	33.3%
Sometimes	5	33.3%	6	40.0%	5	33.3%
Never	4	26.7%	3	20.0%	4	26.7%
Missing	*0*	*0.0%*	*0*	*0.0%*	*0*	*0.0%*
2. Have you ever talked to your child's doctor, nurse, or other health care provider about any kind of health information you have gotten from the internet?						
Yes	11	73.3%	9	64.3%	13	86.7%
No	4	26.7%	5	35.7%	2	13.3%
Missing	*0*	*0.0%*	*1*	*6.7%*	*0*	*0.0%*
3. When you talked with your child's health care provider, how interested were they in hearing about the information you found online? *(of n = 11 people who responded "yes" at baseline to ever talking to provider about info found on internet, n = 9 at three months, and n = 13 at six months)*						
Very interested	1	9.1%	1	11.1%	2	15.4%
Somewhat interested	9	81.8%	6	66.7%	9	69.2%
A little interested	1	9.1%	2	22.2%	2	15.4%
Not at all interested	0	0.0%	0	0.0%	0	0.0%
Missing	*0*	*0.0%*	*0*	*0.0%*	*0*	*0.0%*
4. The following questions are about your communication with your child's doctors, nurses, or other health professionals you saw during the last 12 months. How often did they do each of the following? *(of n = 11 people who responded "yes" at baseline to ever talking to provider about info found on internet, n = 9 at three months, and n = 13 at six months)*						

Give you the chance to ask all the health-related questions you had						
Always	3	27.3%	3	33.3%	5	38.5%
Usually	5	45.5%	4	44.4%	6	46.2%
Sometimes	2	18.2%	2	22.2%	2	15.4%
Never	1	9.1%	0	0.0%	0	0.0%
Missing	*0*	*0.0%*	*0*	*0.0%*	*0*	*0.0%*
Give the attention you needed to your feelings and emotions						
Always	3	27.3%	3	33.3%	3	23.1%
Usually	4	36.4%	2	22.2%	7	53.8%
Sometimes	4	36.4%	4	44.4%	3	23.1%
Never	0	0.0%	0	0.0%	0	0.0%
Missing	*0*	*0.0%*	*0*	*0.0%*	*0*	*0.0%*
Involve you in the decisions about your child's health care as much as you wanted						
Always	3	27.3%	6	66.7%	6	46.2%
Usually	6	54.5%	1	11.1%	5	38.5%
Sometimes	1	9.1%	2	22.2%	2	15.4%
Never	1	9.1%	0	0.0%	0	0.0%
Missing	*0*	*0.0%*	*0*	*0.0%*	*0*	*0.0%*
Make sure you understood the things you needed to do to take care of your child's health						
Always	3	27.3%	4	44.4%	7	53.8%
Usually	5	45.5%	4	44.4%	3	23.1%
Sometimes	2	18.2%	1	11.1%	3	23.1%
Never	1	9.1%	0	0.0%	0	0.0%
Missing	*0*	*0.0%*	*0*	*0.0%*	*0*	*0.0%*
Explain things in a way that you could understand						
Always	3	27.3%	5	55.6%	7	53.8%
Usually	6	54.5%	3	33.3%	3	23.1%
Sometimes	1	9.1%	1	11.1%	3	23.1%
Never	1	9.1%	0	0.0%	0	0.0%
Missing	*0*	*0.0%*	*0*	*0.0%*	*0*	*0.0%*
Spend enough time with you						
Always	3	27.3%	3	33.3%	5	38.5%
Usually	6	54.5%	5	55.6%	6	46.2%
Sometimes	1	9.1%	1	11.1%	2	15.4%
Never	1	9.1%	0	0.0%	0	0.0%

Missing	*0*	*0.0%*	*0*	*0.0%*	*0*	*0.0%*
Help you deal with feelings of uncertainty about your child's health or health care						
Always	3	27.3%	3	33.3%	4	30.8%
Usually	3	27.3%	1	11.1%	5	38.5%
Sometimes	4	36.4%	5	55.6%	4	30.8%
Never	1	9.1%	0	0.0%	0	0.0%
Missing	*0*	*0.0%*	*0*	*0.0%*	*0*	*0.0%*
5. Overall, how would you rate the quality of health care your child received in the past 12 months (baseline survey) or past 3 months (three and six month survey)						
Excellent	3	21.4%	4	26.7%	5	33.3%
Very good	6	42.9%	5	33.3%	7	46.7%
Good	3	21.4%	4	26.7%	3	20.0%
Fair	1	7.1%	2	13.3%	0	0.0%
Poor	1	7.1%	0	0.0%	0	0.0%
Missing	*1*	*6.7%*	*0*	*0.0%*	*0*	*0.0%*
In the past 12 months (baseline survey) or 3 months (three and six month survey), how often did you feel you could rely on your doctors, nurses, or other health care professionals to take care of your child's health care needs?						
Always	5	33.3%	3	21.4%	5	33.3%
Usually	6	40.0%	7	50.0%	8	53.3%
Sometimes	3	20.0%	4	28.6%	2	13.3%
Never	1	6.7%	0	0.0%	0	0.0%
Missing	*0*	*0.0%*	*1*	*6.7%*	*0*	*0.0%*
**Section 5: Participating in the Whole Genome Sequencing Research Study**						
Sometime people feel differently about genetic testing decisions after they receive results. When you think about having gone through the whole genome testing for your child and the results you received, how much would you agree or disagree with the following statements?						
1. It was the right decision						

Strongly agree	15	100.0%	13	86.7%	13	86.7%
Agree	0	0.0%	1	6.7%	1	6.7%
Neither agree nor disagree	0	0.0%	1	6.7%	1	6.7%
Disagree	0	0.0%	0	0.0%	0	0.0%
Strongly disagree	0	0.0%	0	0.0%	0	0.0%
2. I regret the choice that was made						
Strongly agree	0	0.0%	0	0.0%	0	0.0%
Agree	0	0.0%	0	0.0%	0	0.0%
Neither agree nor disagree	0	0.0%	1	6.7%	0	0.0%
Disagree	1	6.7%	1	6.7%	1	6.7%
Strongly disagree	14	93.3%	13	86.7%	14	93.3%
3. I would go for the same choice if I had to do it over again						
Strongly agree	15	100.0%	12	80.0%	14	93.3%
Agree	0	0.0%	1	6.7%	1	6.7%
Neither agree nor disagree	0	0.0%	2	13.3%	0	0.0%
Disagree	0	0.0%	0	0.0%	0	0.0%
Strongly disagree	0	0.0%	0	0.0%	0	0.0%
4. The choice did me a lot of harm						
Strongly agree	0	0.0%	0	0.0%	0	0.0%
Agree	0	0.0%	0	0.0%	0	0.0%
Neither agree nor disagree	0	0.0%	1	6.7%	0	0.0%
Disagree	1	6.7%	1	6.7%	1	6.7%
Strongly disagree	14	93.3%	13	86.7%	14	93.3%
5. The decision was a wise one						
Strongly agree	15	100.0%	13	86.7%	13	86.7%
Agree	0	0.0%	1	6.7%	1	6.7%
Neither agree nor disagree	0	0.0%	1	6.7%	0	0.0%
Disagree	0	0.0%	0	0.0%	0	0.0%
Strongly disagree	0	0.0%	0	0.0%	1	6.7%
Decision Regret Score, median (IQR)	0	(0, 0)	0	(0, 0)	0	(0, 0)
Decision Regret Score						
0	14	93.3%	12	80.0%	12	80.0%
> 0	1	6.7%	3	20.0%	3	20.0%
Decision Regret Score						
Expressed regret on at least one of 5 questions	0	0.0%	2	13.3%	2	13.3%
No regret expressed on any questions	15	100.0%	13	86.7%	13	86.7%

Decision Regret Score for Those with Scores > 0, median (IQR)<br>*n = 1 baseline score of 10*<br>*n = 3 three month scores ranging from 15 to 45*<br>*n = 3 six month scores ranging from 10 to 25*	10	(10, 10)	25	(15, 45)	20	(10, 25)
**Section 6: Response to whole genome sequencing results (only applies to n = 4 respondents of patients with CV)**						
1. I understood how my child came to have this gene						
Strongly agree	2	50.0%	1	25.0%	2	100.0%
Agree	2	50.0%	1	25.0%	0	0.0%
Neither agree nor disagree	0	0.0%	0	0.0%	0	0.0%
Disagree	0	0.0%	0	0.0%	0	0.0%
Strongly disagree	0	0.0%	2	50.0%	0	0.0%
Missing	*0*	*0.0%*	*0*	*0.0%*	*2*	*50.0%*
2. I understand the health risks my relatives face because of my child having the gene						
Strongly agree	2	50.0%	1	25.0%	2	100.0%
Agree	2	50.0%	1	25.0%	0	0.0%
Neither agree nor disagree	0	0.0%	0	0.0%	0	0.0%
Disagree	0	0.0%	0	0.0%	0	0.0%
Strongly disagree	0	0.0%	2	50.0%	0	0.0%
Missing	*0*	*0.0%*	*0*	*0.0%*	*2*	*50.0%*
3. I feel certain that I undertand the meaning of my child having this gene						
Strongly agree	2	50.0%	1	25.0%	0	0.0%
Agree	2	50.0%	1	25.0%	2	100.0%
Neither agree nor disagree	0	0.0%	0	0.0%	0	0.0%
Disagree	0	0.0%	0	0.0%	0	0.0%
Strongly disagree	0	0.0%	2	50.0%	0	0.0%
Missing	*0*	*0.0%*	*0*	*0.0%*	*2*	*50.0%*
4. I understand the chances I have of passing this gene along to other children						
Strongly agree	2	50.0%	2	50.0%	2	100.0%
Agree	2	50.0%	0	0.0%	0	0.0%
Neither agree nor disagree	0	0.0%	0	0.0%	0	0.0%
Disagree	0	0.0%	0	0.0%	0	0.0%

Strongly disagree	0	0.0%	2	50.0%	0	0.0%
Missing	*0*	*0.0%*	*0*	*0.0%*	*2*	*50.0%*
5. I feel that I can explain to other people what my child having this gene means						
Strongly agree	2	50.0%	1	25.0%	0	0.0%
Agree	2	50.0%	1	25.0%	2	100.0%
Neither agree nor disagree	0	0.0%	0	0.0%	0	0.0%
Disagree	0	0.0%	0	0.0%	0	0.0%
Strongly disagree	0	0.0%	2	50.0%	0	0.0%
Missing	*0*	*0.0%*	*0*	*0.0%*	*2*	*50.0%*
PAGIS Certainty Score, median (IQR)	4.5	(4.0, 5.0)	2.6	(1.0, 4.6)	4.6	(4.6, 4.6)
**Section 7: Response to the whole genome sequencing results**						
The statements below reflect responses some people have after learning a child's genetic test result. Please respond how much each of these statements represents how you have felt in the past week. In the past week how often have you been?						
1. Feeling upset about your child's test results?						
Never	10	71.4%	11	73.3%	11	73.3%
Rarely	3	21.4%	3	20.0%	3	20.0%
Sometimes	1	7.1%	1	6.7%	1	6.7%
Often	0	0.0%	0	0.0%	0	0.0%
Missing	*1*	*6.7%*	*0*	*0.0%*	*0*	*0.0%*
2. Feeling sad about your child's test results?						
Never	8	57.1%	13	86.7%	9	60.0%
Rarely	3	21.4%	1	6.7%	4	26.7%
Sometimes	3	21.4%	0	0.0%	2	13.3%
Often	0	0.0%	1	6.7%	0	0.0%
Missing	*1*	*6.7%*	*0*	*0.0%*	*0*	*0.0%*
3. Feeling anxious or nervous about your child's test results?						
Never	10	71.4%	12	80.0%	11	73.3%
Rarely	2	14.3%	2	13.3%	2	13.3%
Sometimes	2	14.3%	0	0.0%	2	13.3%
Often	0	0.0%	1	6.7%	0	0.0%

Missing	*1*	*6.7%*	*0*	*0.0%*	*0*	*0.0%*
4. Feeling guilty about your child's test results?						
Never	10	71.4%	11	73.3%	12	80.0%
Rarely	4	28.6%	2	13.3%	3	20.0%
Sometimes	0	0.0%	2	13.3%	0	0.0%
Often	0	0.0%	0	0.0%	0	0.0%
Missing	*1*	*6.7%*	*0*	*0.0%*	*0*	*0.0%*
5. Feeling relieved about your child's test results?						
Never	6	46.2%	9	60.0%	7	46.7%
Rarely	1	7.7%	3	20.0%	3	20.0%
Sometimes	5	38.5%	2	13.3%	1	6.7%
Often	1	7.7%	1	6.7%	4	26.7%
Missing	*2*	*13.3%*	*0*	*0.0%*	*0*	*0.0%*
6. Feeling happy about your child's test results?						
Never	7	53.8%	10	66.7%	7	46.7%
Rarely	0	0.0%	2	13.3%	4	26.7%
Sometimes	6	46.2%	1	6.7%	3	20.0%
Often	0	0.0%	2	13.3%	1	6.7%
Missing	*2*	*13.3%*	*0*	*0.0%*	*0*	*0.0%*
7. Feeling loss of control						
Never	7	58.3%	11	73.3%	9	60.0%
Rarely	1	8.3%	1	6.7%	1	6.7%
Sometimes	2	16.7%	1	6.7%	5	33.3%
Often	2	16.7%	2	13.3%	0	0.0%
Missing	*3*	*20.0%*	*0*	*0.0%*	*0*	*0.0%*
8. Having problems enjoying your life because of your child's results						
Never	11	84.6%	12	80.0%	12	80.0%
Rarely	1	7.7%	1	6.7%	2	13.3%
Sometimes	0	0.0%	2	13.3%	1	6.7%
Often	1	7.7%	0	0.0%	0	0.0%
Missing	*2*	*13.3%*	*0*	*0.0%*	*0*	*0.0%*
9. Worring about your child's risk of becoming sick or ill						
Never	5	38.5%	4	26.7%	7	46.7%
Rarely	4	30.8%	3	20.0%	5	33.3%
Sometimes	2	15.4%	6	40.0%	1	6.7%
Often	2	15.4%	2	13.3%	2	13.3%
Missing	*2*	*13.3%*	*0*	*0.0%*	*0*	*0.0%*

10. Being uncertain about what your child's results mean about your child's future health						
Never	6	46.2%	6	40.0%	7	46.7%
Rarely	3	23.1%	3	20.0%	2	13.3%
Sometimes	2	15.4%	3	20.0%	4	26.7%
Often	2	15.4%	3	20.0%	2	13.3%
Missing	*2*	*13.3%*	*0*	*0.0%*	*0*	*0.0%*
11. Being uncertain about what your child's results mean for any other children and/or your family's health future						
Never	7	53.8%	12	80.0%	9	60.0%
Rarely	3	23.1%	2	13.3%	4	26.7%
Sometimes	3	23.1%	1	6.7%	1	6.7%
Often	0	0.0%	0	0.0%	1	6.7%
Missing	*2*	*13.3%*	*0*	*0.0%*	*0*	*0.0%*
12. Having difficulty making decisions about health screening or disease prevention for your child						
Never	8	61.5%	10	66.7%	10	66.7%
Rarely	4	30.8%	4	26.7%	3	20.0%
Sometimes	1	7.7%	1	6.7%	2	13.3%
Often	0	0.0%	0	0.0%	0	0.0%
Missing	*2*	*13.3%*	*0*	*0.0%*	*0*	*0.0%*
13. Understanding clearly your child's choices for health screening or disease prevention						
Never	2	15.4%	7	46.7%	5	33.3%
Rarely	3	23.1%	3	20.0%	2	13.3%
Sometimes	2	15.4%	2	13.3%	3	20.0%
Often	6	46.2%	3	20.0%	5	33.3%
Missing	*2*	*13.3%*	*0*	*0.0%*	*0*	*0.0%*
14. Feeling frustrated that there are no definite health guidelines for your child						
Never	2	15.4%	4	26.7%	6	40.0%
Rarely	3	23.1%	1	6.7%	2	13.3%
Sometimes	6	46.2%	7	46.7%	1	6.7%
Often	2	15.4%	3	20.0%	6	40.0%
Missing	*2*	*13.3%*	*0*	*0.0%*	*0*	*0.0%*
15. Thinking about your child's result has affected your work or family life						

Never	10	76.9%	11	73.3%	12	80.0%
Rarely	1	7.7%	2	13.3%	3	20.0%
Sometimes	2	15.4%	1	6.7%	0	0.0%
Often	0	0.0%	1	6.7%	0	0.0%
Missing	*2*	*13.3%*	*0*	*0.0%*	*0*	*0.0%*
16. Feeling concerned about how your child's results will affect your health insurance status						
Never	11	84.6%	14	93.3%	14	93.3%
Rarely	0	0.0%	1	6.7%	1	6.7%
Sometimes	2	15.4%	0	0.0%	0	0.0%
Often	0	0.0%	0	0.0%	0	0.0%
Missing	*2*	*13.3%*	*0*	*0.0%*	*0*	*0.0%*
17. Having difficulty talking about your child's results with family members						
Never	10	76.9%	15	100.0%	14	93.3%
Rarely	2	15.4%	0	0.0%	0	0.0%
Sometimes	1	7.7%	0	0.0%	0	0.0%
Often	0	0.0%	0	0.0%	1	6.7%
Missing	*2*	*13.3%*	*0*	*0.0%*	*0*	*0.0%*
18. Feeling that your family has been supportive during the process of learning the genetic result						
Never	3	23.1%	7	46.7%	5	33.3%
Rarely	2	15.4%	0	0.0%	2	13.3%
Sometimes	2	15.4%	3	20.0%	3	20.0%
Often	6	46.2%	5	33.3%	5	33.3%
Missing	*2*	*13.3%*	*0*	*0.0%*	*0*	*0.0%*
19. Feeling satisfied with family communication about your child's genetic results						
Never	1	7.7%	8	53.3%	6	40.0%
Rarely	2	15.4%	1	6.7%	2	13.3%
Sometimes	2	15.4%	2	13.3%	1	6.7%
Often	8	61.5%	4	26.7%	6	40.0%
Missing	*2*	*13.3%*	*0*	*0.0%*	*0*	*0.0%*
20. Worrying that your child's results have brought about conflict within your family						
Never	11	84.6%	14	93.3%	14	93.3%
Rarely	2	15.4%	1	6.7%	0	0.0%
Sometimes	0	0.0%	0	0.0%	0	0.0%

Often	0	0.0%	0	0.0%	1	6.7%
Missing	*2*	*13.3%*	*0*	*0.0%*	*0*	*0.0%*
21. Feeling regret about getting the test result						
Never	12	92.3%	15	100.0%	15	100.0%
Rarely	1	7.7%	0	0.0%	0	0.0%
Sometimes	0	0.0%	0	0.0%	0	0.0%
Often	0	0.0%	0	0.0%	0	0.0%
Missing	*2*	*13.3%*	*0*	*0.0%*	*0*	*0.0%*
22. Feeling that the genetic test result has made it harder to cope with my child's diagnosis						
Never	10	76.9%	14	93.3%	14	93.3%
Rarely	3	23.1%	1	6.7%	1	6.7%
Sometimes	0	0.0%	0	0.0%	0	0.0%
Often	0	0.0%	0	0.0%	0	0.0%
Missing	*2*	*13.3%*	*0*	*0.0%*	*0*	*0.0%*
23. Feeling that the genetic test result has made it easier to cope with my child's diagnosis						
Never	6	46.2%	9	60.0%	8	53.3%
Rarely	0	0.0%	1	6.7%	1	6.7%
Sometimes	5	38.5%	1	6.7%	3	20.0%
Often	2	15.4%	4	26.7%	3	20.0%
Missing	*2*	*13.3%*	*0*	*0.0%*	*0*	*0.0%*
MICRA Distress Subscale Raw Score, median (IQR) *(n missing baseline = 3)*	2.5	(0.5, 5.0)	0.0	(0.0, 4.0)	0.0	(0.0, 6.0)
MICRA Distress Subscale Scaled Score (all Qs answered), median (IQR) *(n missing baseline = 3)*	8.3	(1.7, 16.7)	0.0	(0.0, 13.3)	0.0	(0.0, 20.0)
MICRA Distress Subscale Scaled Score (at least 1 Q answered), median (IQR) *(n missing baseline = 1)*	5.3	(0.0, 16.7)	0.0	(0.0, 13.3)	0.0	(0.0, 20.0)
MICRA Uncertainty Subscale Raw Score, median (IQR) *(n missing baseline= 2)*	6.0	(2.0, 11.0)	8.0	(1.0, 12.0)	7.0	(0.0, 14.0)
MICRA Uncertainty Subscale Scaled Score, median (IQR) *(n missing baseline= 2)*	13.3	(4.4, 24.4)	17.8	(2.2, 26.7)	15.6	(0.0, 31.1)

MICRA Uncertainty Subscale Scaled Score (at least 1 Q answered), median (IQR)<br>*(n missing baseline= 2)*	13.3	(4.4, 24.4)	17.8	(2.2, 26.7)	15.6	(0.0, 31.1)
MICRA Positive Experience Subscale Raw Score, median (IQR)<br>*(n missing baseline = 2)*	10.0	(4.0, 12.0)	14.0	(9.0, 20.0)	11.0	(6.0, 16.0)
MICRA Positive Experience Subscale Scaled Score, median (IQR)<br>*(n missing baseline = 2)*	50.0	(20.0, 60.0)	70.0	(45.0, 100.0)	55.0	(30.0, 80.0)
MICRA Positive Experience Subscale Scaled Score (at least 1 Q answered), median (IQR)<br>*(n missing baseline = 2)*	50.0	(20.0, 60.0)	70.0	(45.0, 100.0)	55.0	(30.0, 80.0)
MICRA Overall (Qs 1-21) Raw Score, median (IQR)<br>*(n missing baseline = 3)*	23.5	(16.0, 27.5)	25.0	(21.0, 35.0)	24.0	(20.0, 26.0)
MICRA Overall (Qs 1-21) Scaled Score, median (IQR)<br>*(n missing baseline = 3)*	22.4	(15.2, 26.2)	23.8	(20.0, 33.3)	22.9	(19.0, 24.8)
MICRA Overall (Qs 1-21) Scaled Score (at least 1 Q answered), median (IQR)<br>*(n missing baseline = 1)*	20.5	(9.0, 25.7)	23.8	(20.0, 33.3)	22.9	(19.0, 24.8)
**Section 8: Questions about the report you received on your child's genetic testing**						
1. Did you read the letter describing your child's whole genome sequencing results?						
Yes	13	86.7%	12	92.3%	6	40.0%
No	2	13.3%	1	7.7%	9	60.0%
Missing	*0*	*0.0%*	*2*	*13.3%*	*0*	*0.0%*
2. How helpful were the various parts of the genetic testing report you received? *(of n = 13 at baseline, n = 12 at three months, and n = 6 at six months who said they read the letter)*						
The summary						
Not helpful	0	0.0%	0	0.0%	0	0.0%
A little bit helpfu	0	0.0%	1	10.0%	1	16.7%
Somewhat helpful	2	20.0%	2	20.0%	2	33.3%
Quite a bit helpful	4	40.0%	2	20.0%	3	50.0%
Very helpful	4	40.0%	5	50.0%	0	0.0%

Missing	*3*	*23.1%*	*2*	*16.7%*	*0*	*0.0%*
The detailed explanation						
Not helpful	0	0.0%	0	0.0%	0	0.0%
A little bit helpfu	0	0.0%	2	20.0%	1	16.7%
Somewhat helpful	2	20.0%	2	20.0%	2	33.3%
Quite a bit helpful	5	50.0%	1	10.0%	3	50.0%
Very helpful	3	30.0%	5	50.0%	0	0.0%
Missing	*3*	*23.1%*	*2*	*16.7%*	*0*	*0.0%*
The care instructions						
Not helpful	0	0.0%	0	0.0%	0	0.0%
A little bit helpfu	0	0.0%	1	11.1%	2	33.3%
Somewhat helpful	2	22.2%	2	22.2%	2	33.3%
Quite a bit helpful	5	55.6%	1	11.1%	2	33.3%
Very helpful	2	22.2%	5	55.6%	0	0.0%
Missing	*4*	*30.8%*	*3*	*25.0%*	*0*	*0.0%*
3. Did you review the genetic testing report you received with your provider? *(of n = 13 at baseline, n = 12 at three months, and n = 6 at six months who said they read the letter)*						
Yes, in person	8	72.7%	11	91.7%	3	50.0%
Yes, over the phone	0	0.0%	0	0.0%	0	0.0%
No	3	27.3%	1	8.3%	3	50.0%
Missing	*2*	*15.4%*	*0*	*0.0%*	*0*	*0.0%*
4. Did reviewing the genetic testing report with your provicer help improve your understanding of your child's health condition? *(of n = 8 at baseline, n = 11 at three months, and n = 3 at six months who said they read the letter with their provider)*						
n/a No, I already understood everything I needed	1	12.5%	0	0.0%	0	0.0%
Didn't help	1	12.5%	2	18.2%	1	33.3%
Helped a little	0	0.0%	1	9.1%	1	33.3%
Helped some	1	12.5%	1	9.1%	0	0.0%
Helped a fair amount	0	0.0%	3	27.3%	1	33.3%
Helped a great deal	5	62.5%	4	36.4%	0	0.0%
Missing	*0*	*0.0%*	*0*	*0.0%*	*0*	*0.0%*
